# Life-Time Environmental Chemical Exposure and Obesity: Review of Epidemiological Studies Using Human Biomonitoring Methods

**DOI:** 10.3389/fendo.2021.778737

**Published:** 2021-11-11

**Authors:** Nayan Chandra Mohanto, Yuki Ito, Sayaka Kato, Michihiro Kamijima

**Affiliations:** Department of Occupational and Environmental Health, Nagoya City University Graduate School of Medical Sciences, Nagoya, Japan

**Keywords:** environmental chemicals, phthalates, persistent organic pollutants, overweight, obesity, bisphenols, environmental obesogens, human biomonitoring

## Abstract

The exponential global increase in the incidence of obesity may be partly attributable to environmental chemical (EC) exposure. Humans are constantly exposed to ECs, primarily through environmental components. This review compiled human epidemiological study findings of associations between blood and/or urinary exposure levels of ECs and anthropometric overweight and obesity indices. The findings reveal research gaps that should be addressed. We searched MEDLINE (PubMed) for full text English articles published in 2006–2020 using the keywords “environmental exposure” and “obesity”. A total of 821 articles were retrieved; 102 reported relationships between environmental exposure and obesity indices. ECs were the predominantly studied environmental exposure compounds. The ECs were grouped into phenols, phthalates, and persistent organic pollutants (POPs) to evaluate obesogenic roles. In total, 106 articles meeting the inclusion criteria were summarized after an additional search by each group of EC combined with obesity in the PubMed and Scopus databases. Dose-dependent positive associations between bisphenol A (BPA) and various obesity indices were revealed. Both individual and summed di(2-ethylhexyl) phthalate (DEHP) and non-DEHP metabolites showed inconsistent associations with overweight and obesity indices, although mono-butyl phthalate (MBP), mono-ethyl phthalate (MEP), and mono-benzyl phthalate (MBzP) seem to have obesogenic roles in adolescents, adults, and the elderly. Maternal exposure levels of individual POP metabolites or congeners showed inconsistent associations, whereas dichlorodiphenyldichloroethylene (DDE) and perfluorooctanoic acid (PFOA) were positively associated with obesity indices. There was insufficient evidence of associations between early childhood EC exposure and the subsequent development of overweight and obesity in late childhood. Overall, human evidence explicitly reveals the consistent obesogenic roles of BPA, DDE, and PFOA, but inconsistent roles of phthalate metabolites and other POPs. Further prospective studies may yield deeper insights into the overall scenario.

## Introduction

Obesity is characterized by excess body fat, total body fat, or a particular depot of body fat (1). The most commonly evaluated anthropometric indices of obesity are body mass index (BMI), waist circumference (WC), hip circumference (HC), skinfold thickness (ST), percent body fat (%BF), fat mass (FM), and waist-to-height ratio (WHtR) (2–5). An adult individual is overweight if BMI ≥25 kg/m^2^ to <30 kg/m^2^, and obese if BMI ≥30 kg/m^2^ or WC ≥80 cm in women and WC ≥90 cm in men (6). Childhood overweight and obesity can be defined as BMI z-scores >1 and >2, respectively (3, 4, 6). Sex- and age-specific WC ≥90^th^ percentile or WHtR ≥0.5 are also used to determine obesity in children (7, 8). Some alternative measurements are still available for both children and adults, and differ with age, gender, and country (9).

Whether obesity should be declared a disease is controversial (1). However, obesity leads to many aspects of ill health or functional impairment and several diseases (10–13), reduces health quality of life (14, 15), and increase mortality and morbidity (16–18). It is a complex condition with many causal contributors, including genetic factors and environmental factors (19–21). Recent epidemiological research has also reported the associations with overweight and obesity of environmental exposure sources that include environmental chemicals (ECs), air pollution, particulate matter, heavy metals, noise, green space, and others (22–31). According to the “obesogen hypothesis,” ECs, which are termed environmental obesogens (EOs), regulate lipid metabolism and adipogenesis, leading to obesity (32).

Over time, the use of synthetic chemicals has grown exponentially with the development of commerce and industry (33). Excessive usage results in environmental contamination. Humans are exposed to these ECs through environmental media by ingestion, inhalation, absorption, and even through transplacental transfer and breast milk (34–42). The human exposure levels of these ECs are generally estimated by biomonitoring of their metabolites or parent compounds in human urine or blood (cord blood or peripheral blood) as exposure biomarkers worldwide (43–47).

Recently, there has been increased interest in epidemiological studies of EC biomonitoring and subsequent evaluation of their obesogenic effects (4, 8, 34, 48–51). A concise view of the overall epidemiological findings is required to clarify whether obesogenic evidence of ECs is sufficient or consistent for the advancement of future research. Some previous reviews have explored the obesogenic role of ECs. However, most of these considered only a single group of ECs, and/or selected ECs based on their endocrine-disrupting properties, and/or considered limited exposure and outcome assessment period or age, and even not focused on epidemiological studies, and/or focused on a mechanism (52–59).

A further review addressing the current epidemiological evidence of the obesogenic effects of ECs at all stages of life from a public health perspective is needed. Accordingly, the objectives of the present review are to illuminate epidemiological study findings of the associations between EC exposure and anthropometric overweight and obesity indices, uncover the current research gap, and contemplate future research.

## Methods

### Selection and Grouping of EOs

**Figure 1:**
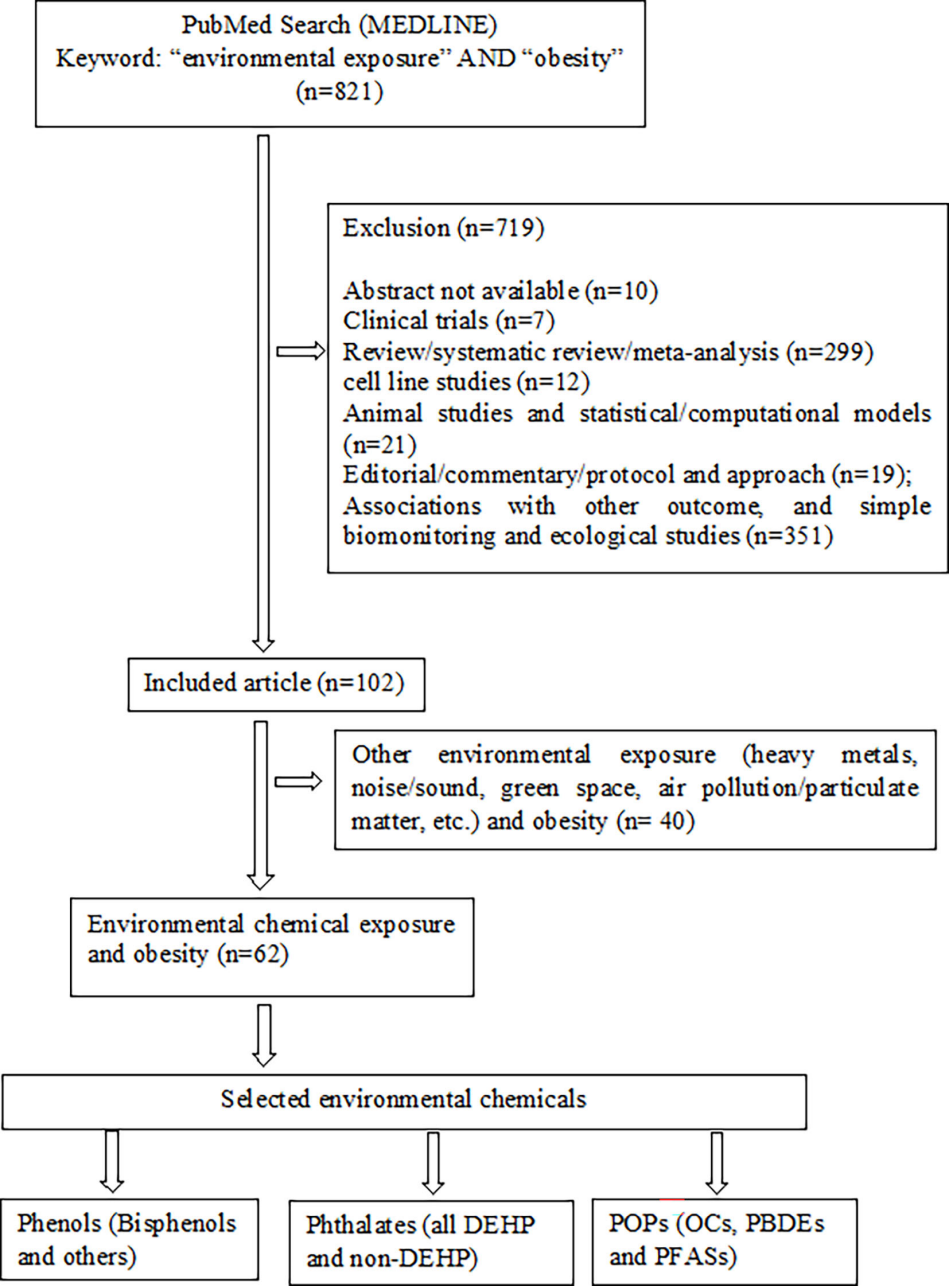
Schematic diagram of the strategy for selection and grouping of environmental obesogens.

Research articles that demonstrated the associations between environmental exposure and obesity in MEDLINE of PubMed were searched for using “environmental exposure” AND “obesity” as keywords to select EOs (**Figure 1**). After additional filtering for full text, journal articles, inclusion of humans, English, and publication year (2006–2020), a total of 821 articles were retrieved. Of these, 719 articles were excluded owing to the following reasons: abstract not available (n=10); involved clinical trials (n=7), review/systematic review/meta-analysis (n=299); cell line studies (n=12); animal studies and statistical/computational models (n=21); editorial/commentary/protocol and approach (n=19); investigated associations of EC exposure with other adverse outcomes, including hypertension, puberty, diabetes, polycystic ovary syndrome, cardiovascular diseases, cancer risk, and others, and simple biomonitoring and ecological studies (n=351). In the remaining 102 articles, the ECs were predominantly studied environmental exposure (ECs = 62 and others = 40). Also, the production and uses of agricultural, industrials, and other synthetic chemicals are increasing, and recognized as major environmental pollutants over other environmental exposures namely heavy metals, noise or sound, green space and particulate matters. Therefore, we selected ECs as the major EOs apart from other environmental exposure and grouped them as follows: (i) phenols [bisphenol A (BPA), bisphenol S (BPS), bisphenol F (BPF), and others], (ii) phthalates (all phthalates and their metabolites), and (iii) persistent organic pollutants (POPs) [organochlorine compounds (OCs), polybrominated diphenyl ethers (PBDEs) and per- and polyfluoroalkyl substances (PFASs), and their metabolites or congeners] (**Figure 1**).

### Literature Search and Inclusion Criteria

**Figure 2:**
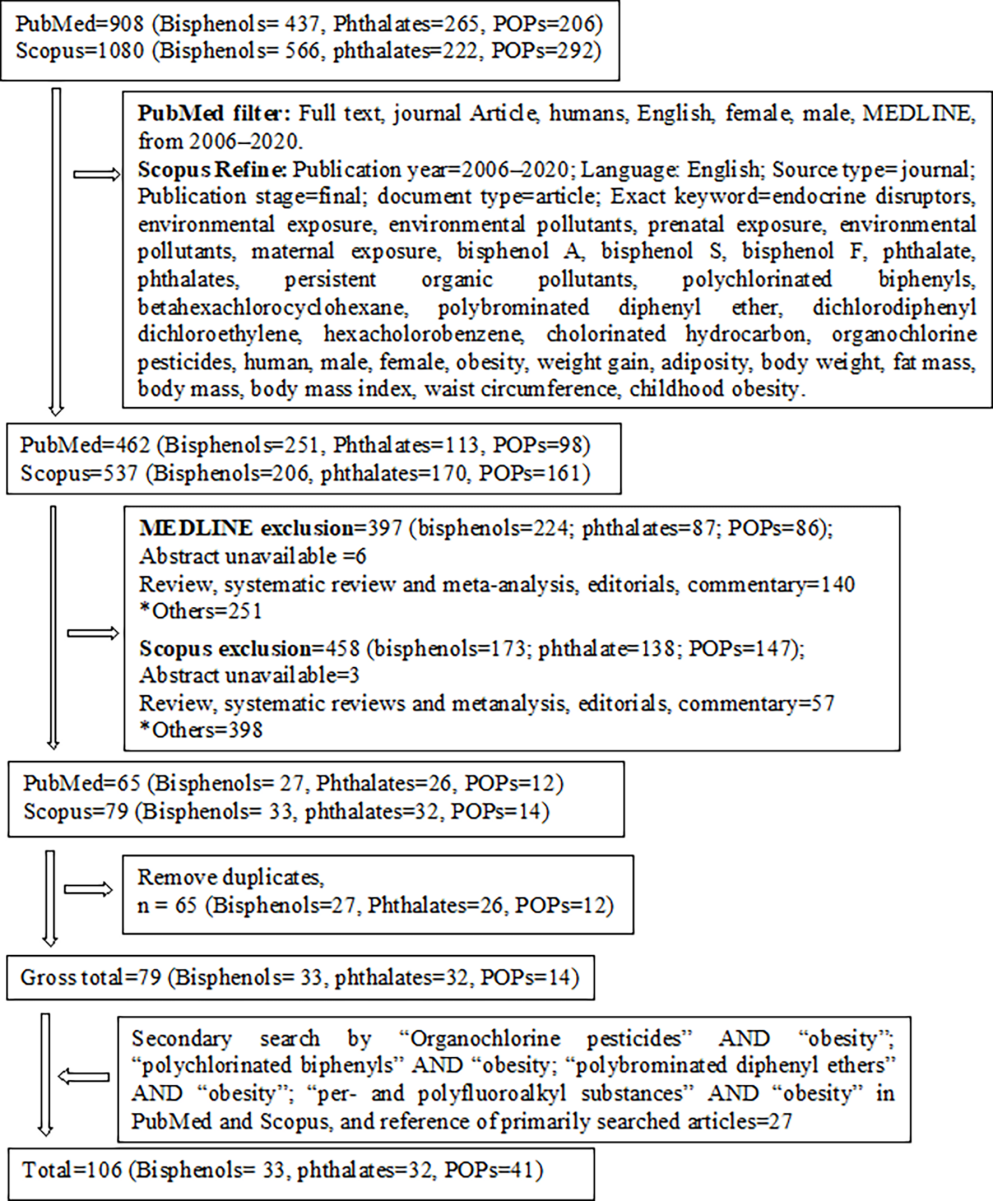
Schematic diagram of study selection. *Cell line studies, animal/rodent/drosophila studies, investigation of other associations (e.g., growth, metabolic syndrome, fatty liver disease, diabetes, cardiometabolic risk, inflammation, polycystic ovary syndrome, prostate cancer, food intake, semen quality, puberty), ecological studies, and or simple biomonitoring studies.

A primary search in PubMed and Scopus databases for each group of EO used the keywords “bisphenols” AND “obesity,” “phthalate” AND “obesity”, and “persistent organic pollutants” AND “obesity” to identify original research articles of human epidemiological studies. Additional PubMed filtering and Scopus refining were performed to select relevant articles (**Figure 2**). Articles were considered relevant when they investigated the associations of selected EOs with anthropometric overweight and obesity indices. The references of the selected primary research articles were also searched for relevant publications. A secondary search was also performed for each group of POPs combined with obesity (**Figure 2**).

All full-length articles, short communications, and brief reports of original research work from all over the world, irrespective of sex, religion, and race/ethnicity, were included in this review (**Figure 1**). Inclusion criteria included (i) epidemiological study (cohort, cross-sectional, and case-control); (ii) all ages and/or life-stage at exposure or outcome assessment; (iii) primary outcomes of overweight and/or obesity, or at least one anthropometric index of overweight or obesity; (iv) EO concentrations measured in urine or blood as human biomonitoring; (v) assessment of only non-occupational exposure levels of EOs; (vi) published after postulating “obesogen hypothesis”; and (vii) written in English. All other articles were excluded (**Figure 2**). Finally, 106 original research articles were included in this review.

### Visualizing Evidence

Associations of EOs with overweight and/or obesity have been demonstrated in the aforementioned three groups. We grouped the early- and later-life exposure and outcome assessment age into seven categories (**Matrix Tables 1–6**): infants (up to 1 year), toddlers (>1– 2 years), preschoolers (>2– 5 years), school-aged (>5– 13 years), adolescents (>13– 19 years), adults (≥20– 60 years), and elderly (>60 years) as classified previously (60). Matrix tables were created according to categories.

## Results

### Environmental Phenols and Obesity

We summarized a total of 33 human epidemiological studies, including 13 cohort studies and 20 cross-sectional studies that explored the association between prenatal and early- to later-life urinary phenols, especially bisphenol exposure levels with anthropometric overweight and obesity indices (**Table 1** and **Matrix Table 1**). Most of the cohort studies were birth cohorts. The study subjects enrolled in the birth cohorts ranged from 173 to 1128 mother-child pairs. Among the 20 cross-sectional studies, 9 involved children and adolescents between the ages of 3 and 19 years, and 11 involved adults and elderly participants >18 years. Both the cohort and cross-sectional studies measured BPA, BPS, and BPF in spot urine other than the first morning void urine, or 24 h urine.

**Table 1:** Associations of environmental phenols with anthropometric overweight and obesity indices.

Ref.	Study type (country),Subjects (n)	Exposure		Outcome ass. time	Covariates	Key findings
		Marker	Biomonitoring time			
(8)	Birth cohort (China), Mother-child (430)	BPA	40 (mean) GW, 3 y, 7 y	7 y	1a, 2a, 3a, 4a, 5a, 6a, 7a, 16a	Maternal urinary BPA concentration (range: 0.17–280 µg/l) was associated with WC in children aged 7 y [β=0.51 (0.07, 0.95)]. Positive associations were observed only in girls [β=0.69 (0.04, 1.34)] not in boys. Risk of AO related to prenatal BPA exposure was higher in the T2 and the T3 than those in the T1 [OR=2.51 (1.15, 5.50) and OR=2.58 (1.19, 5.63), respectively]. No significant associations with GO were evident at 7 y. Risk of AO at 7 y in T3 of early childhood (3 y) BPA exposure was higher than those in T1 [OR=2.86 (1.02, 8.04)].
(2)	Cross-sectional (Korea), Adults (702)	BPA	40.1 y (mean)	40.1y	1, 2, 7, 8, 9a	Urinary BPA levels were not associated with GO risk. Participants in the Q4 of BPA level had 1.75 times higher risk of AO than participants in the Q1 of BPA level. Urinary BPA level significantly associated with AO in women [OR=1.50 (1.00, 2.26)] but not in men [OR=1.13 (0.85, 1.50)]. Also, the association was significant in postmenopausal women [OR=2.23 (1.01, 4.92)] but non-significant in premenopausal women [OR=1.31 (0.78, 2.20)].
(50)	Birth cohort (Netherlands), Mother-child (1128)	BPA, BPS, BPF	1^st^–3^rd^ T_r_	10 y	1ab, 2a, 3a, 5a, 6c, 7b, 8, 10a, 11a	Null associations were evident between maternal bisphenol concentrations and childhood adiposity measures at 10 y.
(61)	Birth cohort (Canada), Mother-child (719)	BPA	6.3–15 GW	1.9–6.2 y	1b, 3a, 4a, 5a, 7b, 10a, 12, 13a	A 2-fold increase in BPA concentrations (range: 0.1–63 ng/ml) was associated with higher waist to hip ratio [β=0.003 (0.001, 0.005)] among overall child. A 2-fold increase in BPA concentrations was associated with increased WC [β=0.20 (0.00, 0.50)] and subscapular ST [β=0.15 (0.01, 0.30)] in girls. Associations were null in boys.
(48)	Cross-sectional (USA), Adults (1269)	BPA, BPS, 2,4-DP, 2,5-DP	≥20 y	≥20 y	1, 2, 3, 4b, 6b, 7, 9b, 10a, 16a	BPS was associated with both GO [OR=1.44 (1.01, 2.07)] and AO [OR=1.47 (1.01, 2.16)] (Q4 *vs*. Q1) whereas BPA showed nonsignificant associations with GO [OR=1.53 (0.99, 2.35)] and AO [OR=1.36 (0.87, 2.13)]. There were no associations of 2,4-DP and 2,5-DP with GO and AO.
(62)	Cross-sectional (Iran), Children, adolescents (132)	BPA	6–18 y	6–18 y	1, 2, 9b, 16a	The mean BMI increased significantly from T1 to T3 (T1: 8.70–78.90, T2: 82.70–246.80 and T3: 247–725 µg/l); [T2: difference=3.65 (1.92, 5.38) and T3: difference=8.26 (6.48, 10.03) *vs* T1, respectively]. Similarly, consistent association also found between BPA levels and WC [7.97 (3.64, 12.31) and 16.26 (11.81, 20.72) at T2 and T3, respectively]. Participants in the T2 and T3 had higher odds for obesity [OR=4.11 (1.56, 10.81) and OR=12.48 (3.36, 46.39), respectively], in comparison with T1.
(63)	Cross-sectional (USA), Children (NA)	BPA	6 y, 19 y	6 y, 19 y	1, 2, 3a, 4bc, 6b, 9c, 10a, 16a	Increase odds of obesity were found among the Q2 [OR=1.25 (0.95, 1.65)], Q3 [OR=1.39 (1.03, 1.86)] and Q4 [OR=1.43 (1.11, 1.84)] compared with Q1 before creatinine adjustment. After the adjustment, the associations were null; Q2 [OR=0.83 (0.66, 1.03)], Q3 [OR=0.91 (0.70, 1.18)] and Q4 [OR=0.95 (0.74, 1.21)]. Children enrolled in 2003–2008 with higher urinary BPA concentrations had elevated odds of obesity, whereas these associations were inconsistent who enrolled during 2009–2014.
(34)	Cross-sectional (USA), Children, adolescents (1831)	BPA, BPS, BPF	6–19 y	6–19 y	1, 2,3, 4b, 10a	A 10-fold increase in BPS, the odds of GO increased by 16% [OR=1.16 (1.02, 1.32)], severe obesity by 18% [OR=1.18 (1.03, 1.35)], and AO by 13% [OR=1.13 (1.02, 1.27)]. Detected BPF (detected *vs* not detected) concentration was associated with an increased prevalence of AO [OR=1.29 (1.01, 1.64)] and continuous BMIZ [β=0.10 (0.01, 0.20)]. BPA was not associated with obesity.
(7)	Cross-sectional (USA), Children, adolescents (745)	BPA, BPF, BPS	6–17 y	6–17 y	1, 2, 4b, 6bd, 9c, 10a,16a	The OR of GO comparing the Q4 with Q1 of urinary bisphenol levels were [1.74 (0.92, 3.31)] for BPA (3.98 *vs* 0.46 ng/ml), [1.54 (1.02, 2.32)] for BPF (1.55 *vs* 0.14 ng/ml), and [1.36 (0.53, 3.51)] for BPS (1.30 *vs* 0.07 ng/ml). Urinary BPA, BPF, and BPS levels (Q4) were significantly associated with both GO and AO only in girls. The weighted prevalence of GO and AO were 21% (15.5, 26.4) and 35% (28.2, 41.9).
(64)	Cross-sectional (Canada), Adults (4733)	BPA	18–79 y	18–79 y	1, 2, 8, 10a, 16a, 6ef	Urinary BPA concentrations was associated with increased odds of GO [OR=1.54 (1.002, 2.37)] and AO [OR=1.16 (0.81, 1.66)] in the Q4 (>2.4 µg/l) *vs*. Q1(<0.7 µg/l). For the overweight category, associations were generally positive but nonsignificant [OR=1.14 (0.73, 1.77)] in the Q4 (*vs*. Q1) of BPA concentrations. A 2.71-fold increase in urinary BPA concentration was associated with increased BMI and WC [β=0.33 (0.10, 0.57)] and [β=1.00 (0.34, 1.65)], respectively.
(65)	Cross-sectional (Korea), Female (296)	BPA	30–49 y	30–49 y	1, 7, 8, 14a	Urinary BPA levels were associated with BMI [β=0.04 (0.01, 0.06)] and WC [β=0.02 (0.01, 0.03)] before the adjustment. BPA levels were also associated with BMI and WC [β=0.03 (0.01, 0.06) and β=0.02 (0.01, 0.03), respectively] even after potential covariate adjustment.
(66)	Cross-sectional (USA), Children (1860)	BPA	8–19 y	8–19 y	1, 3b, 4b, 5b, 6b, 7c, 9cd, 10a, 16ab	Urinary BPA concentration was associated with percentage of trunk fat in girls [β=2.85 (0.92, 4.78) in Q2 (1.50–3.16 ng/ml), [β=2.57 (0.28, 4.85)] in Q3 (3.17–6.05 ng/ml) and [β=2.79 (0.44, 5.14)] in Q4 (≥6.06 ng/ml), compared with Q1 (0.30–1.49 ng/ml). BPA levels in Q4 were associated with elevated LBMI z-score in boys (*p*<0.05), and with elevated FMI z-scores in girls (*p*<0.05). FMI z-scores were increased in the Q2 [β=0.29 (0.06, 0.52)], Q3 [β=0.30 (0.02, 0.57)], and Q4 [β=0.29 (0.04, 0.55)] of urinary BPA concentrations in overall participants.
(67)	Cross-sectional (USA), Adults (1709)	BPA, BPF, BPS	≥20 y	≥20 y	1, 2, 3, 4b, 6b, 7, 9b, 10a, 16a	The OR for GO comparing the Q4 (>2.6, >1.00 and 1.00 ng/ml) with Q1 (<0.6, <0.14 and <0.2 ng/ml) for BPA, BPF and BPS were [1.78 (1.10, 2.89)], [1.02 (0.70, 1.47)], and [1.22 (0.81, 1.83)], respectively. The corresponding odds for AO for BPA, BPF and BPS were [1.55 (1.04, 2.32)], [1.05 (0.68, 1.63)] and [1.16 (0.72, 1.88)], respectively.
(68)	Cohort (USA), Girls (1017)	BPA, 2,5-DP, Triclosan, enterolactone	6–8 y	7–15 y	1, 10a	Positive associations were found between 2,5-DP and BMI, WC and %BF. Enterolactone was inversely associated with changes in BMI, WC, and %BF fat in different ages. Differences in adiposity measurements were observed between tertiles of 2,5-DP (T2 *vs* T1 and T3 *vs* T1) beginning at age 8–9 y, which consistently increased through age 13 y. Triclosan was positively associated with all adiposity measures only among overweight girls. BPA was inversely associated with %BF.
(69)	Cohort (China), Adults (888)	BPA	≥40 y	≥44 y	1, 2, 3, 5c, 7, 8, 14b, 16a,	A 10-fold increase in BPA concentrations was positively associated with 2.30 folds of risk of AO incidence [OR=2.30 (1.39, 3.78)]. Compared with the T1 (0.15–0.48 ng/ml) of urinary BPA concentration, T2 (0.71–1.00 ng/ml) and T3 (1.51–2.95 ng/ml) were associated with a higher risk of AO incidence [OR=1.79 (1.08, 2.97) and OR=1.83 (1.09, 3.08), respectively]. A 10-fold increase in BPA concentration was associated with 1.17 cm increment in WC (SE=0.46, *p*=0.01). BPA positively associated with the AO incidence in women but not in men.
(70)	Birth cohort (Mexico), Mother-child (249)	BPA	3^rd^ T_r,_ 4 y	8–14 y	1ab, 2a, 5a, 3c	Prenatal BPA exposure was not associated with obesity indices. In girls 4 y of age, increased BPA exposure was associated with sum of ST [β=3.47 (0.05, 6.40)]. Child sex modified the relationships between specific gravity-corrected and ln-transformed urinary BPA levels and BMIZ [β=0.05 (−0.16, 0.25)] and sum of ST [β=0.97 (−1.01, 2.94)]. These associations might depend on pubertal transitions.
(71)	Birth cohort (USA), Mother-child (173)	BPA, 2,5-DP, BeP-3, Triclosan	3^rd^ T_r_	4–9 y	1ab, 2a, 3a, 5ade, 6a, 7b, 9b, 10a, 15, 16c	Before adjustment, maternal urinary concentrations of 2,5-DP were associated with greater %FM [β=1.24 (0.08, 2.40)] and BeP-3 were associated with lower %FM [β=−1.13 (−2.24, 0.00)] among children. BeP-3 concentrations were inversely associated with %FM in girls [β=−1.51 (−3.06, 0.01)] but not boys [β=−0.20 (−1.69, 1.26)]. After adjustment, null associations were observed for all phenol markers with %FM.
(72)	Birth cohort (USA), Mother (375) children (408 & 518)	BPA	34 (mean) GW, 3, 5y	5 y, 7 y	2a, 5efg, 10a, 11b, 16cd,	Prenatal ln-transformed BPA concentrations were associated with FMI [β=0.31 (0.01, 0.60)], %BF [β=0.79 (0.03, 1.55)] and WC [β=1.29 (0.29, 2.30)] but null with BMIZ at 7 y. In girls, prenatal urinary BPA concentrations were associated with FMI [β=0.48 (0.50–0.91)] but not in boys at 7 y. Child urinary BPA concentrations (3y, 5 y) were not associated with obesity indices.
(73)	Birth cohort (Greece), Mother-child (500)	BPA	1^st^ T_r_, 2.5 y, 4 y	2.5 y, 4 y	1b, 2a, 3a, 5ag, 6a, 15	BPA concentrations at 4 y were associated with increased child BMIZ [β=0.2 (0.01, 0.4)], WC [β=1.2 (0.1, 2.2)] and sum of ST [β=3.7 (0.7, 6.7)], and a higher prevalence of obesity [RR=2.9 (0.8, 10.5)] at age 4. Log_10_-transformed creatinine-adjusted BPA concentrations during pregnancy and early childhood (2.5 y and 4 y) were associated with obesity [RR=0.1 (0.003, 5.4) for maternal; RR= 0.3 (0.01, 6.4) and RR= 2.9 (0.8, 10.5) for childhood BPA, respectively].
(74)	Panel-cohort (Korea), Elder people (558)	BPA	60–87 y	60–87 y	1, 2, 6bg, 7c, 8, 9a, 14bc	Per IQR increase (0.96 μg/g of creatinine) in log-transformed BPA was associated with overweight [OR=1.17 (1.04, 1.32)]. A significant association was found in women [OR=1.25 (1.09, 1.45)], but not in men [OR=0.97 (0.77, 1.22)]. ORs of overweight increased with quartiles of BPA (Q1 = 0.38, Q2 = 0.39–0.75, Q3 = 0.76–1.41 and Q4=≥1.42 µg/g of creatinine) in women [Q2 OR=1.54 (1.02, 2.32), Q3 OR=1.70 (1.10, 2.62), and Q4 OR=1.81 (1.13, 2.92)].
(75)	Cross-sectional (Cyprus), Adults (223)	BPA, mono-chloro BPA	≥18 y	≥18 y	1, 2, 3, 14d	A significant correlation was observed between creatinine-adjusted urinary mono-chloro (mCl) BPA and BMI (r_S_=0.18, *p*=0.0087). Observed an increase prevalence in above normal BMI participants with increasing tertile of creatinine-adjusted urinary ln-transformed mClBPA (*p*=0.056) but not for BPA (*p*=0.254). An increase in the OR for above normal BMI was observed for the T3 of creatinine-adjusted urinary BPA [>2697 ng/g, OR=1.17(0.57, 2.43)] and mClBPA [>108 ng/g, OR=1.14(0.50, 2.59)] compared with T1.
(76)	Cross-sectional (Italy), Elder male (76)	BPA	53.5 y (mean)	53.5 y	NA	Significantly higher BPA levels were observed in the subjects with visceral obesity (WC>102 cm) compared to the subjects with WC<102 cm.
(77)	Cross-sectional (Korea), Adults (1030)	BPA	44.3 y (mean)	44.3 y	1, 2, 3, 4a, 7, 8, 16a	WC was higher among subjects with a urinary BPA concentration in the Q4 (>2.594 µg/ml) relative to those in the Q1 (<0.853 µg/ml) (*p*=0.0071). Positive associations were found between urinary BPA concentrations and BMI (β=0.1866; *p*=0.0128), WC (β=0.0564; *p*=0.0533), and %BF (β=0.1091; *p*=0.0389). Subjects at Q4 were more likely to be obese compared to those at Q1 [OR=1.94 (1.31, 2.86)].
(78)	Birth cohort (USA), Mother-child (297)	BPA	2^nd^–3^rd^ T_r_, 1–2 y	2–5 y	1b, 3a, 4ac, 5c, 6h, 7c, 10a, 11a, 12, 14e	A 10-fold increase in prenatal and early-childhood BPA concentrations was associated with a reduction in child BMI [β=−0.1 (−0.5, 0.3) and β=−0.2 (−0.6, 0.1), respectively]. Children in the early-childhood at T3 of BPA (20–314 μg/g of creatinine) had lower BMI at 2 y [difference=−0.3 (−0.6, 0.0)] and larger increases in their BMI slope from 2 through 5 y [BMI increase per year=0.12 (0.07, 0.18)] than children in the T1 (2.1–11 μg/g of creatinine) [BMI increase per year=0.07 (0.01, 0.13)].
(79)	Birth cohort (USA), Mother-child (311)	BPA	1^st^ –2^nd^ T_r_, 5 y, 9 y	5 y, 9 y	6, 8, 9, 10, 43, 44, 45 3a, 4a, 5a, 7b, 6ij, 13b	Prenatal BPA concentrations was associated with decreased BMIZ [β=−0.47 (−0.87, −0.07)] and %BF [β=−4.36 (−8.37, −0.34)] and decreased odds of overweight/obesity [OR=0.37 (0.16, 0.91)] in T3 (1.7–27.0 µg/l) *vs* T1 (<LOD–1.0 µg/l) among girls. Urinary BPA concentrations at 5 y of age were not associated with obesity indices at 5 or 9 y. BPA concentrations at 9 y were positively associated with BMIZ [β=0.55 (0.15, 0.95)], WC [β=5.89 (1.19, 10.59)], FM [β=4.62 (0.26, 8.98)], and overweight/obesity [β=4.20 (1.60, 11.02)] at 9 y in boys and girls.
(80)	Birth cohort (Spain), Mother-child (402)	BPA	1^st^, 3rd T_r_	6 m, 14 m, 4 y	1ab, 2a, 3a, 5a, 7b, 10b, 11ac	A 10-fold increase in creatinine adjusted BPA concentration was associated with increased WC z-score [β=0.28 (0.01, 0.57)], BMIZ [β=0.28 (−0.06, 0.63)], and BMIZ ≥85th percentile [RR=1.38 (0.72, 2.67)] at 4 y. BPA was not associated with obesity-related outcomes at earlier ages (at 6 m and 14 m of age).
(81)	Cross-sectional (China), School children (1326)	BPA	9–12 y, ≥ 12 y	9–12 y, ≥ 12 y	1, 2, 3a, 5h, 6ek, 9ef, 13c, 14a, 16a	A higher urine BPA level (≥2 µg/l) was associated with more than 2-fold increased risk of overweight/obese (weight ≥90th percentile) among girls aged 9–12 y [OR=2.32 (1.15, 4.65)]. Similar associations were also found for hip circumference [OR=2.88 (1.12, 7.45)], WC [OR=2.60 (0.98, 6.91)], weight to height ratio [OR=2.38 (0.92, 6.16)], ST [OR=1.86 (0.73, 4.71)] and BMI [OR=1.47 (0.71, 3.05)]. A dose-response relationship was evident between urinary BPA level [<50^th^ (BPA conc.<0.98 µg/ml, ref.), 50^th^ to 75^th^ {BPA conc. = 0.98–4.13 µg/ml, OR=1.92 (0.79, 4.66)}, 75^th^ to 90^th^ {BPA conc. = 4.13–10.04 µg/ml, OR=2.04 (0.77, 5.41)}, >90^th^ percentile {BPA conc.>10.04 µg/ml, OR=5.18 (1.68, 15.91)}] and overweight.
(82)	Cross-sectional (USA), Children (2664)	BPA	6–18 y	6–18 y	1, 2, 3, 7c, 9b, 10a, 16a	Compared with children in the Q1 of BPA (<1.5 ng/ml), children in the Q4 (>5.4 ng/ml) had a higher odd for BMI [OR=1.17 (0.50, 1.84)] and for obesity [OR=2.55 (1.65, 3.95)]. Positive association with obesity was predominantly present in boys [OR=3.80 (2.25, 6.43)] and in non-Hispanic whites [OR=5.87 (2.15, 16.05)].
(83)	Cross-sectional (USA), Children and adolescents (2838)	BPA	6–19 y	6–19 y	1, 2, 3a, 4b, 6b, 7c, 9c, 10a, 16a	Urinary BPA showed dose-dependent associations with BMIZ. The odds of obesity were increased in the Q2 [OR=2.22 (1.53, 3.23)], Q3 [OR=2.09 (1.48, 2.95)], and Q4 [OR=2.53 (1.72, 3.74)] of urinary BPA concentration. Children in the Q1 BPA (<1.5 ng/ml) had a lower prevalence of obesity [10.3% (7.5, 13.1)] than those in Q2 (1.5–2.7 ng/ml) [20.1% (14.5, 25.6)], Q3 (2.8–5.5 ng/ml) [19.0% (13.7, 24.2)], and Q4 (≥5.6 ng/ml) [22.3% (16.6, 27.9)]. Race/ethnicity-urinary BPA quartile interaction with obesity as the outcome showed significant interactions for only non-Hispanic white participants [Q2 OR=3.10 (1.33, 7.21); Q3 OR=3.33 (1.48, 7.49); Q4 OR=4.08 (1.66, 10.0)].
(84)	Cross-sectional (China), Adults (3390)	BPA	≥40 y	≥40 y	1, 2, 3, 7, 8, 14cfg, 16a,	Compared with the participants in the Q1, those in the Q4 of urinary BPA had significantly higher BMI (*p*<0.001) and WC (*p*<0.001). Observed highest prevalence of GO [OR=1.50 (1.15, 1.97)] and AO [OR=1.28 (1.03, 1.60)] in the Q4 of BPA (>1.43 ng/ml) in compared with Q1 (≤0.47 ng/ml), Q2 (0.48–0.81 ng/ml) and Q3 (0.82–1.43 ng/ml).
(85)	Cross-sectional (China), Children (259)	BPA	8–15 y	8–15 y	1, 2, 16d	Log-transformed urinary BPA concentrations were significantly associated with increasing BMI [β=0.017 (0.002, 0.032)] in all subjects.
(86)	Cross-sectional (USA), Adults (3967)	BPA	≥20 y	≥20 y	1, 2, 3, 7, 8, 9b, 10a, 14bhi	Positive association was found between increasing levels of urinary BPA, and both GO and AO. Compared with Q1 (<1.10 ng/ml), Q4 (>4.20 ng/ml) had higher odds for GO [OR=1.69 (1.30, 2.20) and AO [OR=1.59 (1.21, 2.09)] in whole population. Similar associations were also found after stratification in men, women, non-Hispanic white, non-Hispanic blacks and Mexican Americans and others (*p <*0.05).
(41)	Cross-sectional (USA), Adults (2747)	BPA	18–74 y	18–74 y	1, 2, 3, 7, 10a, 16a	Compared to participants in the Q1 of BPA (≤1.1 ng/ml), participants in the Q4 were obese [{Q2 (1.2–2.3 ng/ml), OR=1.85 (1.22, 2.79)}; {Q3 (2.4–4.6 ng/ml), OR=1.60 (1.05, 2.44)}; {Q4 (≥4.7 ng/ml), OR=1.76 (1.06, 2.94)}]. Higher BPA concentration was also associated with AO [Q2 OR=1.62 (1.11, 2.36); Q3 OR=1.39 (1.02, 1.90); Q4 OR=1.58 (1.03, 2.42)].

n, number; y, year; m, month; T_r_, trimester; Q, quartile/quantile; T, tercile/tertile; NA, not available; GO, general obesity; AO, abdominal/central obesity; BPA, bisphenol A; BPS, bisphenol S; BPF, bisphenol F; DP, dichlorophenol; BeP-3, benzophenone-3; IQR, interquartile range; BMI, body mass index; BMIZ, BMI z-score; WC, waist circumference; BF, body fat; FM, fat mass; FMI, fat mass index; LBMI, lean body mass index; ST, skinfold thickness; OR, odds ratio; RR, relative risk; GW, weeks of gestation; ∑DEHP, sum of di-2-ethylhexyl phthalate.

^1^age (^a^child age, ^b^maternal age); ^2^sex (^a^child sex); ^3^education level (^a^maternal/paternal education, ^b^caregivers education, ^c^mother years of schooling); ^4^socioeconomic status (^a^household/family income, ^b^poverty to income ratio, ^c^insurance status); ^5^physique (^a^pre-pregnancy/maternal BMI, ^b^height, ^c^BMI, ^d^gestational weight gain, ^e^maternal height, ^f^pre-pregnancy obesity); ^6^food (^a^breast feeding, ^b^total energy/calorie intake, ^c^maternal diet quality score, ^d^alternative healthy eating index, ^e^eating junk food, vegetables or fruit, ^f^suger-sweetend beverage consumption, ^g^fatty acid intake, ^h^food security during pregnancy, ^i^soda consumption during pregnancy, ^j^child fast food and sweet consumption at 9 y, ^k^unbalanced diet); ^7^smoking (^a^child’s passive smoking, ^b^smoking during pregnancy, ^c^serum/urinary cotinine); ^8^alcohol consumption; ^9^activity (^a^regular exercise, ^b^regular exercise, ^c^TV/video watching time, ^d^computer use, ^e^sports/activities, ^f^playing video games); ^10^race (^a^maternal/paternal/child race/ethnicity, ^b^maternal country of origin); ^11^information of pregnancy (^a^parity, ^b^gestational age, ^c^time of day of urine collection in the 1st and 3rd trimester); ^12^maternal marital status; ^13^location of participants or study (^a^study Centre, ^b^years of USA residence, ^c^residence); ^14^past history (^a^depression score, ^b^diabetes, ^c^lipid profile, ^d^health status, ^e^depressive symptoms, ^f^systolic blood pressure, ^g^C-reactive protein, fasting plasma glucose, insulin, alanine aminotransferase and gamma-glutamyl transferase, ^h^hypertension, ^i^TC); ^15^work status during pregnancy; ^16^others (^a^urinary creatinine level, ^b^survey year, ^c^maternal/prenatal sum of DEHP, ^d^urinary specific gravity).

[All outcome ranges within the first bracket indicate the 95% CI].

Maternal urinary BPA levels showed null or positive associations with one or more anthropometric obesity indices in infants and toddlers (61, 73, 80). Similar associations were also found between maternal BPA exposure levels and obesity measures in preschoolers and school-aged children. These associations were sex-specific (8, 50, 61, 70–73, 78, 80). Only one study reported negative associations between prenatal BPA exposure and BMI z-score and %BF (79). Toddler and preschooler exposure levels of BPA reported null or positive associations with overweight or obesity indices in toddlers, preschoolers, and school-aged children (8, 70, 72, 73, 78, 79). Associations were mostly null in children 5 to 9 years of age (72, 79). Urinary BPA concentrations among school-aged children showed inconsistent relationships with one or more obesity indices (8, 63, 68, 79, 81). However, several studies recruited children with ages ranging from 6 to 19 years and investigated the associations of urinary BPA, BPS, and BPF exposure levels with overweight and obesity indices. All these studies found positive associations with one or more anthropometric parameters of obesity (7, 34, 62, 66, 82, 83, 85). Adult exposure levels of BPA, BPS, and BPF were also positively associated with at least one anthropometric index of obesity in adults and elderly individuals (2, 41, 48, 64, 65, 67, 69, 75–77, 84, 86) with the exception of inconsistent associations in one study (63). One panel study (cohort) investigated the association between urinary BPA concentrations and overweight. The authors reported a positive association in the case of overall and female study participants, but not in male participants (74). Some other studies also observed a sex-stratified relationship between prenatal bisphenol exposure and overweight and obesity indices (8, 61, 72, 79). A few studies reported sex-dependent associations between childhood bisphenol exposure levels and obesity or adiposity measures (7, 66, 81). Race- or ethnicity-specific associations of urinary BPA concentrations with obesity indices were also reported, with a significant association of BPA levels only in non-Hispanic white subjects (83). Pubertal status was reported as a confounder of the associations between BPA concentrations and BMI, WC, and ST, especially in girls (70, 79). Maternal exposure levels of 2, 5-dichlorophenol, benzophenone-3, and triclosan showed null associations with %FM in children aged 4–9 years (71). In contrast, one study reported positive associations between urinary 2, 5-dichlorophenol levels in children aged 6–8 years and BMI, WC, and %BF in later childhood, which consistently increased up to 13 years of age (68).

BPA levels in urine varied among the studies and ranged from non-detectable to >2594 ng/ml (**Table 1**). Children and adolescents (6–19 years) with urinary BPA, BPS, and BPF concentrations of ≥2, ≥1.30, and ≥0.2 ng/ml, are susceptible to developing overweight or obesity (7, 34, 62, 81). In adults, BPA, BPS, and BPF showed obesogenic effects at concentrations ≥0.71, ≥1, and 1 ng/ml, respectively (41, 64, 67, 69, 77, 86). In addition, BPA concentrations ≥0.39 ng/ml may be responsible for subsequent development of overweight or obesity in elderly people (74).

### Environmental Phthalates and Obesity

A total of 32 studies (11 birth cohort, 19 cross-sectional, and 2 case-control studies) explored the association of both prenatal and postnatal urinary exposure levels of phthalate metabolites with overweight and obesity measures in human populations of different ages (**Table 2**, and **Matrix Tables 2**, **3**). In the birth cohort studies, urine samples were collected from both the pregnant mother and their children aged 1–14 years. The study subjects ranged from 128 to 1128 mother-child pairs in the birth cohorts. Among the 19 cross-sectional studies, 11 involved children and adolescents, 8 involved only adults and elderly people (male and/or female) of different ages. Almost all the studies determined phthalate metabolites in the spot urine of the study participants.

**Table 2:** Associations of environmental phthalates with anthropometric overweight and obesity indices.

Ref.	Study type (country),Subjects (n)	Exposure		Outcome ass. time	Covariate	Key findings
		Markers	Biomonitoring time			
(49)	Cross-sectional (China) Elder men and women (942)	MBP, MEP, MMP, DEHP (MEHP, MEHHP, MEOHP), MBzP	≥60 y	≥60 y	1, 2, 4a, 7, 8, 9a, 13a, 15	Increased urinary concentrations of MEOHP [Q4 OR=1.93 (1.33, 2.78)], MBP [Q2 OR=1.67 (1.16, 2.41); Q3 OR=2.31 (1.60, 3.35); Q4 OR=3.24 (2.22, 4.72)], MEP [Q3 OR=1.90 (1.32, 2.74); Q4 OR=2.10 (1.45, 3.03)], and MMP [Q2 OR=1.63 (1.13, 2.35); Q3 OR=1.81 (1.25, 2.60); Q4 OR=2.38 (1.14, 3.44)] were correlated with higher odds of GO. Urinary MBP levels were also associated with AO [Q2 OR=1.93 (1.21, 3.07), Q3 OR=2.42 (1.47, 3.99), and Q4 OR=2.31 (1.38, 3.88) *vs* Q1]. In men, increased concentrations of MBP [Q2 OR=2.64 (1.50, 4.62), Q3 OR=3.16 (1.69, 5.89), and Q4 OR=2.77 (1.39, 5.49)] were correlated with AO. No significant associations were observed in women.
(2)	Cross-sectional (Korea), Adult (702)	MiBP, MBP, MECPP MEHHP, MEOHP, MBzP	40.1 y	40.1 y (mean)	1, 2, 7, 8, 9b	Log transformed urinary phthalate metabolite concentrations were not associated with GO [∑Phthalate metabolites, OR=0.93 (0.68, 1.28)] and AO [∑Phthalate metabolites, OR=0.98 (0.68, 1.40)].
(50)	Birth cohort (Netherland), Mother-child (1128)	MiBP, MBnP, MEP, MMP, MBzP, MHxP, MHpP, MCHP, MCPP MEHHP, MEOHP, MECPP, MCMHP, PA	1^st^ –3^rd^ T_r_	10 y	1ab, 2a, 3a, 5a, 6a, 7, 8, 10a 11a	A 2.72-fold increase in PA concentrations in 1^st^ T_r_ of pregnancy were associated with an increase in childhood BMI [SDS=0.07 (0.00, 0.14)]. Null associations were observed between other phthalate metabolites and BMI.
(87)	Birth cohort (Mexico), Mother-child (223)	MBP, MiBP, MEP, MCPP, MEHP, MEHHP, MEOHP, MECPP, MBzP	1^st^ –3^rd^ T_r_, 8–14 y	8–14 y, 9–17 y	1b, 3a, 18a	Natural log-transformed 1^st^ T_r_ MiBP concentrations were associated with increased ST [β=3.41 (1.50, 5.31)], BMIZ [β=0.28 (0.12, 0.45)] and WC [β=2.33 (0.86, 3.8)], and MBP with only BMIZ [β=0.25 (0.03, 0.46)]. Second T_r_ MBzP concentration was associated with decreased ST [β=−2.53 (−4.78, −0.28)] among girls. Maternal urinary 2^nd^ T_r_ MBzP concentration was also associated with BMIZ [β=0.25 (0.01, 0.49)] and WC [β=2.11 (0.27, 3.95)] among boys.
(88)	Cross-sectional (Korea), Adults (4752)	MBP, ∑DEHP (MEHHP, MEOHP, MECPP), MBzP	≥19 y	≥19 y	1, 3, 12, 4a, 7, 8, 9b	In women, urinary MEHHP and ∑DEHP concentrations were associated with obesity [Q4 *vs* Q1; OR=1.72 (1.19, 2.49) and OR=1.52 (1.04, 2.21), respectively]. In men, urinary MBP concentration was found to be significantly associated with obesity [Q4 *vs* Q1; OR=0.71 (0.50, 0.99)]. Women ≥50 y showed positive associations between the MEHHP, MEOHP, ∑DEHP, and MBzP concentrations and obesity [Q4 *vs* Q1; OR=1.94 (1.28, 2.94), OR=1.88 (1.21, 2.94), OR=2.04 (1.31, 3.18), and Q3 *vs* Q1; OR=1.45 (1.02, 2.05)], respectively.
(48)	Cross-sectional (USA), Adults (1269)	MEP, MCOP, MECPP	≥20 y	≥20 y	1, 2, 3, 4a, 6b, 7, 9a, 10a, 18b,	MCOP concentrations were associated with BMI [β=0.36 (0.06–0.66)] and WC [β=0.98 (0.28, 1.69)]. MCOP and MECPP were associated with GO [OR=1.80 (1.22, 2.65) and OR=1.62 (1.04, 2.51)], and AO [OR=1.70 (1.14, 2.54) and OR=1.59 (1.01, 2.51)] at Q4 *vs* Q1. The weighted quantile sum index was associated with both GO [OR=1.63 (1.21, 2.20)] and AO [OR=1.66 (1.18, 2.34)].
(89)	Cross-sectional (Iran), Children, adolescent (242)	MBP, MEHP, MMP, MEOHP, MEHHP, MBzP,	6–18 y	6–18 y	1, 2, 9a, 14abc, 17	All metabolites had significant positive association with BMI, whereas only MEOHP showed a significant association with WC after the adjustment. Most of the phthalates were associated with obesity (T3 *vs* T1) [MBP: OR=1.26 (0.54, 1.98)], [MBzP: OR=5.54 (4.79, 6.28)], [MMP: OR=4.26 (3.56, 4.96)], [MEHP: OR=3.63 (2.95, 4.31)], and [MEHHP: OR=4.16 (3.31, 5.01)].
(90)	Case-control (Korea), Girls (137)	MEHP, MEHHP, MEOHP, MECPP	6–13 y	6–13 y	1, 5b, 16a	In pubertal girls, null associations were found between DEHP metabolites and obesity indices. %MEHHP among all DEHP metabolites was higher in the overweight prepubertal girls than in the controls. The %MEHHP was positively associated with the BMI [β=1.93 (0.18, 3.70)], WC [β=0.67 (0.15, 1.19)], and %BF [β=0.60 (0.03, 1.18)] in prepubertal girls. The %MEHHP of prepubertal girls in Q3 and Q4 was significantly higher OR for AO than those in Q1 (OR=5.05 for Q3 and OR=7.30 for Q4).
(91)	Case-control (China), Child (149)	MMP, MEP, MBP, MEHP, MEOHP, MEHHP	10–15 y	10–15 y	1, 2, 4b, 6b, 9a, 16b	Compared with normal weight children, higher levels of MBP were detected in urinary samples of children with overweight and obesity. Positive association was found between urinary MBP concentration and childhood overweight/obesity [OR=1.586 (1.043, 2.412)].
(92)	Cross-sectional (Iran), Child (242)	MBP, MEHP, MMP, MEOHP, MEHHP, MBzP	6–18 y	6–18 y	1, 2, 18c	After adjustment, all metabolites showed a positive relationship with BMIZ (β=0.17 for MEOHP, β=0.18 for MBzP, β=0.22 for MBP, β=0.23 for MEHP and β=0.30 for MEHHP; *p ≤* 0.005) and WC (β=0.14 for MMP, β=0.19 for MEOHP, β=0.22 for MBzP, β=0.29 for MBP, β=0.37 for MEHP and β=0.39 for MEHHP; *p ≤* 0.02).
(93)	Birth cohort (Greece), Mother-child (260 & 500)	MBP, MiBP, MEP, MEHP, MEHHP, MEOHP, MBzP	1^st^ Tr, 4 y	4–6 y	1ab, 2a, 3a, 5a, 7a, 11a	A 10-fold increase in maternal ΣDEHP was associated with decreased WC [β=−2.6 (−4.72, −0.48)] in boys and [β=2.14 (−0.14, 4.43) in girls and WHtR [β=−0.01 (−0.03, 0.01)] in boys and [β=0.02 (0.01, 0.04) in girls. Child MEP and MBP were associated with lower BMIZ in boys [β=−0.22 (−0.44, −0.01) and β=−0.1 (−0.35, −0.15), respectively] and with higher BMIZ in girls [β=0.17 (−0.12, 0.45) and β=0.39 (0.11, 0.66), respectively]. Child ΣDEHP showed similar associations in boys *vs* girls. A 10-fold increase in child MiBP was associated with a change in BMIZ [β=−0.31 (−0.6, −0.02)] in boys and [β=0.74 (0.37, 1.1)] in girls.
(94)	Cross-sectional (China), Children (276)	MBP, MEP, MMP, MEHP, MEOHP, MEHHP, MECPP, MCPP, MOP, MCOP, MBzP	6–8 y	6–8 y	2, 3a, 13ab, 18a,	In boys, a 1 ng/ml increase in MBzP, MECPP, or MEOHP level was associated with decreased WC [β=-0.011 (−0.021, −0.001), β=−0.023 (−0.038, −0.007), or β=−0.010 (−0.019, −0.001), respectively]. In girls, a 1 ng/ml increase in MMP concentrations was associated with increased ST [β=0.039 (0.002, 0.076)], whereas MECPP concentrations were associated with decreased ST [β=−0.050 (−0.095, −0.005). A 1 ng/ml increase in the MEHP level was associated with decreased BMI [β=−0.020 (−0.036, −0.005)].
(70)	Birth cohort (Mexico), Mother-child (249)	MBP, MEP, MiBP, MEHP, MEHHP, MECPP, MEOHP, MCPP, MBzP	1^st^ T_r_	8–14 y	1ab, 2a, 3a, 5a	Prenatal MBzP concentration was inversely associated with BMIZ [β=−0.21 (−0.41, −0.02)] and child urinary MEHP concentration was inversely associated with WC [β=−1.86 (−3.36, −0.35)] and ΣST [β=−2.08 (−3.80, −0.37)]. Child urinary phthalate metabolites were showed significant inverse relationship with BMIZ [MEOHP, β=−0.26 (−0.51, −0.005)], WC [MECPP, β=−2.13 (−4.22, −0.04); MEHHP, β=−2.02 (−4.02, −0.03) and MEOHP, β=−2.13 (−4.16, −0.10)], and ΣST [MEHP, β=−2.95 (−5.08, −0.82)] in boys but all associations were null in girls.
(95)	Birth cohort (USA), Mother-child (537 & 345)	MBP, MiBP, MEP, DEHP (MEHP, MEHHP, MEOHP, MECPP), MCPP, MCOP, MCNP, MBzP	14 and 26.9 GW (mean)	5–12 y	1b, 3a, 4b, 6c, 7a, 12, 13c, 18d	Log_2_-transformed prenatal urinary metabolites of DEP, DBP, BzBP, and DEHP were positively associated with BMIZ, WCZ, and %BF at 5, 7, 9,10.5, and 12 y. A 2-fold increase in prenatal concentrations of some metabolites were associated with increased odds of being overweight/obese [MEP, OR=2.0 (1.0, 3.9), MBP, OR=2.1 (1.1, 4.2), MBzP, OR=1.9 (0.9, 3.7), and ΣDEHP, OR=2.2 (1.1, 4.5)] at 12 y. A 2-fold increase in MBP was associated with a change in BMIZ of 0.13 (0.02, 0.24) in boys *vs* −0.01 (−0.14, 0.12) in girls and a change in WCZ of 0.10 (0.01, 0.20) in boys *vs* −0.03 (−0.15, 0.08) in girls.
(65)	Cross-sectional (Korea), Female (296)	MBP, MEHHP, MEOHP	30–49 y	30–49 y	1, 7, 8, 14a	Null associations were found between urinary MEHHP, MEOHP and MBP concentrations, and BMI and WC [MEHHP, β=0.03 (−0.01, 0.06) and β=0.002 (−0.01, 0.02); MEOHP, β=0.02 (−0.02, 0.05) and β=−0.001 (−0.01, 0.01); and MBP, β=−0.01 (−0.04, 0.02) and β=−0.002 (−0.01, 0.01)].
(96)	Birth cohort (USA), Mother-child (219)	MiBP, MBnP, MEP, DEHP (MEHP, MEHHP, MEOHP, MECPP), MCPP MBzP	16, 26 GW (mean), 1–4 y, 5, 8 y	8 y	1ab, 2a, 3a, 4ac, 5a, 6de, 7, 10a, 11a, 12, 14d	Both maternal and childhood urinary MBzP concentrations were inversely associated with adiposity at 8 y of age. A 10-fold increase in prenatal urinary MBzP concentrations was associated with reduction in BF [β=−1.7 (−3.6, −0.2) at age 8 y. A 10-fold increase in ∑DEHP concentrations at 1 and 5 y was associated with decrease [β=−2.7 (−4.8, −0.5)] and increase [β=2.9 (0.3, 5.5)] in %BF, respectively. MEP concentrations at 8 y of age were associated with higher child adiposity, but earlier childhood concentrations were not.
(97)	Cross-sectional (China), Adults (2330)	MBP, MiBP, MMP, MEP, MEHP, MEOHP, MEHHP, MECPP, MCMHP, MBzP	>18 y	>18 y	1, 2, 3, 6bf, 7, 12	Overall, higher urinary levels of MMP, MBzP, MEHHP, and MECPP were associated with increased OR of AO. Significant increased odds were observed in Q2 [OR=1.56 (1.11, 2.20)], Q3 [OR=1.33 (1.05, 1.88)], and Q4 [OR=1.91 (1.34, 2.72)] of MMP; [OR=1.52 (1.18, 2.06)] of MBzP; Q2 [OR=1.46 (1.13, 1.90)], Q3 [OR=1.53 (1.18, 1.98)], and Q4 [OR=1.56 (1.19, 2.04)] of MEHHP; and Q3 [OR=1.43 (1.11, 1.84)] and Q4 [OR=1.33 (1.02, 1.74)] of MECPP. Higher urinary levels of MMP, and MEHHP were associated with increased odds of AO in females in Q2 [OR=1.79 (1.16, 2.75)], Q3 [OR=1.59 (1.04, 2.42)], and Q4 [OR=2.02 (1.33, 3.06)] of MMP; and Q2 [OR=1.63 (1.04, 2.54)], Q3 [OR=2.37 (1.51, 3.72)], and Q4 [OR=1.80 (1.16, 2.81)] of MEHHP.
(98)	Birth cohort (USA), Mother-child (404 & 180)	DEP, MEP, MBP, MiBP, ∑DEHP (MEHP, MEHHP, MEOHP, MECPP), MCPP MBzP,	3^rd^ T_r_	4–9 y	1ab, 2a, 3a, 5abc, 6g, 7a, 9a, 10a. 15a, 18e	Maternal ∑DEHP concentrations were associated with decreased %FM [β=−3.06 (−5.99, −0.09)] among children in the T3 of ∑DEHP concentrations than in the children in T1. Null associations were evident between ∑DEHP metabolite concentrations and BMIZ.
(99)	Birth cohort (USA), Mother-child (707)	MiBP, MBP, MEP, ∑DEHP (MEHP, MEHHP, MEOHP, MECPP), MBzP, MCPP	2^nd^–3^rd^ T_r_	4–9 y	1b, 2a, 3a, 5abc, 6g, 7a, 10a, 11a, 13d, 15a, 18be	Prenatal urinary MCPP concentrations were positively associated with overweight/obese status in children [OR=2.1 (1.2, 4.0)] but not with BMIZ [β=−0.02 (−0.15, 0.11)]. Maternal MEP and ∑DEHP concentrations showed negative trend with BMIZ among girls [β=−0.14 (−0.28, 0.00) and β=−0.12 (−0.27, 0.02), respectively]. Urinary MCPP concentrations of Hispanic in compared with non-Hispanic blacks showed higher odds [OR=3.7 (1.6, 9.1)] of being overweight/obese, although had null association with BMIZ [β =0.08 (−0.11, 0.27).
(100)	Birth cohort (Korea) Mother-child (128)	∑DEHP (MEHHP, MEOHP)	38–40 GW	3 m	1b, 5a, 11bc, 18b	∑DEHP exposure levels in newborns were associated with decrease of ponderal index in boys (β=−0.13, *p*=0.021). ∑DEHP metabolites concentrations in newborns’ urine were also associated with increased BMIZ during the 3 m after birth [OR=4.35 (1.20, 15.72)].
(101)	Birth cohort (USA), Mother-child (424)	MiBP, MBnP, MEP, MEHP, MEHHP, MEOHP, MECPP, MCPP, MBzP	3^rd^ T_r_, 3 y, 5 y	5, 7 y	1a, 4d, 5a, 10a, 15a, 18a	In PCA analysis, prenatal DEHP component scores were non significantly and inversely associated with BMIZ at 5 and 7 y. In boys, higher maternal non-DEHP component scores were associated with lower BMIZ [β=−0.30 (−0.50, −0.10)], %BF [β=−1.62 (−2.91, −0.34)], FM index [β=−0.50 (−0.96, −0.04) and smaller WC [β=−2.02 (−3.71, −0.32)] at 7 y.
(102)	Cross-sectional (USA), Girls (1239)	LMWP (MEP, MBP, MiBP, DBP, DiBP), ΣDEHP (MEHP, MEOHP, MECPP, MEHHP), MBzP, MCPP	6–8 y	7–13 y	1, 1c, 10a, 18fgh	LMWP were positively associated with gains in BMI and WC and differences between girls with high (≥194 μg/g cr) *vs* low (<78 μg/g cr) concentrations increased from 0.56 (−0.02, 1.1) to 1.2 (0.28, 2.1) and from 1.5 (−0.38, 3.3) to 3.9 (1.3, 6.5), respectively from 7 to 13 y. Null associations were found for HMWP (ΣDEHP, MBzP and MCPP) or ΣDEHP with BMI or WC differences.
(103)	Birth cohort (Spain), Mother-child (470)	MiBP, MBP, MEP, MEHP, MEHHP, MECPP, MEOHP, MCMHP, MBzP, 7OHMMeOP	1^st^, 3^rd^ T_r_	7 y	1ab, 2a, 4b, 5acd, 6g, 7a, 10b, 11b	For 7OHMMeOP, T3 estimates were decreased compared to T1 [β=−0.29 (−0.59, 0.01)]. Maternal phthalate metabolites concentrations were negatively associated with BMIZ and overweight but significant associations was found between phthalate metabolites and overweight at T2 *vs* T1 [RR=0.49 (0.26, 0.94)].
(104)	Cross-sectional (USA), Female (1690)	MBP, MEP, MEHP, MEHHP, MEOHP, MECPP, MBzP	≥18 y	≥18 y	1, 3, 4b, 6b, 7, 8, 9a, 6f, 10a, 16c	MBP concentrations were associated with BMI and WC [OR=1.13 (1.03, 1.23) and OR=1.13 (1.03, 1.24), respectively], and MEHP with only BMI [OR=1.12 (1.03, 1.23)]. A higher ratio of MEHP to MEHHP was associated with BMI [OR=1.21 (1.09, 1.34)] and WC [OR=1.20 (1.10, 1.31)].
(105)	Cross-sectional (Taiwan), Adolescents (270)	LMWP (MMP, MEP, MiBP, MBP) MEHP, MEHHP, MECPP, MEOHP, MBzP	6.5–15 y	6.5–15 y	1, 2, 18b	MEP, MiBP, MEOHP, MEHHP, MECPP, and LMWP were positively associated with WC; MEP, MEOHP, MEHHP and LMWP were positively associated with ST; MEP, MiBP, MEOHP, MEHHP, MECPP, LMWP, and PAEs were positively associated with WHtR; MEP, MiBP, MBP, MEOHP, MEHHP, LMWP, and total phthalate metabolites were positively associated with waist to hip ratio, and MEP and MEHHP were positively associated with BMI. Indices (except HC) significantly increased among general adolescents with 25–75^th^ and >75^th^ percentile of phthalate metabolites in compared with <25^th^ percentile.
(106)	Cross-sectional (USA), Children, adolescents and adults	LMWP (MBP, MiBP, MEP), HMWP (MEHP, MECPP, MEHHP, MEOHP, MBzP, MCNP, MCOP)	6–19 y, ≥20 y	6–19 y, ≥20 y	1, 2, 3, 4a, 6b, 7, 7b, 8, 9c, 10a, 14e, 18b	LMWP were associated with higher odds [Q2 (0.27–0.52 µmol/ml), OR=3.97 (2.23, 7.08); Q3 (0.53–1.10 µmol/ml), OR=3.13 (1.69, 5.81); and Q4 (>1.10 µmol/ml), OR=5.39 (1.87, 15.53) *vs* Q1 (≤0.26 µmol/ml)] for obesity in male children and adolescents. Similar associations were also found for overall children and adolescents. HMWP and ∑DEHP metabolites were associated with higher odds for obesity [Q3 OR=1.59 (1.19, 2.13) and Q4 OR=1.77 (1.26, 2.48) for HMWP, and for ΣDEHP, Q4 OR=1.62 (1.11, 2.37) *vs* Q1] in all adults.
(107)	Cross-sectional (China), Children (493)	LMWP (MEP, MBP, MMP), ∑MEHP (MEHP, MEOHP, MEHHP)	8–13 y	8–13 y	4b, 6b, 9a, 16b	MBP and LMWP were positively associated with obesity in boys in a concentration-effect manner. In the 11–13 y group, LMWP level was positively associated with all obesity indices, including subscapular ST, WC and HC, %BF, BMI, BMIZ, and BSA. The Q3 and Q4 of MBP were significantly associated with higher BSA, BMI, BMIZ, subscapular ST and HC. In girls, inverse associations were found between urinary MEHP, MEHHP and ∑MEHP and obesity (*p*=0.05).
(108)	Cross-sectional (USA), Children (2884)	LMWP (MEP, MBP, MiBP), HMWP (MEHP, MECPP, MEHHP, MEOHP, MBzP)	6–19 y	6–19 y	1, 2, 3a, 4b, 6b, 7b, 9d, 18b	A 2.72-fold increase in LMWP metabolites was associated with increased odds of overweight and obesity [OR=1.21 (1.05, 1.39)] and [OR=1.22 (1.07, 1.39)], respectively, and increased BMIZ [β=0.09 (0.003, 0.18)], among non-Hispanic blacks. MEP was associated with BMIZ [β=0.08 (0.01–0.16)], overweight [β=1.18 (1.04, 1.34)] and obesity [β=1.19 (1.05, 1.35)] among non-Hispanic blacks. HMWP or DEHP metabolites had null associations.
(109)	Cross-sectional (China), School children (259)	LMWP {∑DBP (MBP, MiBP), MMP, MEP}, HMWP {∑DEHP (MEHP, MECPP, MEHHP, MEOHP, MCMHP), MOP, MiNP, MCHP, MBzP}	8–15 y	8–15 y	1, 2	The log-transformed concentrations of nine urine phthalate metabolites and five molar sums (sum of DEHP, LMWP, HMWP, DBP and all metabolites) were positively associated with BMI or WC after the adjustment for age and sex. Only MEHP and MEP showed significant positive association with BMI [β=0.048 (0.007, 0.089) and β=0.022 (0.005, 0.040)] and WC [β=0.038 (0.006, 0.071) and β=0.020 (0.006, 0.033)] after additional adjustment of other phthalate concentrations as covariates.
(110)	Cross-sectional (USA), Children (387)	LMWP (MEP, MBP, MiBP) HMWP (MECPP, MEHHP, MEOHP, MEHP, MBzP), MCPP	6–8 y	6–8 y	1, 2, 3a, 6b, 9ef, 10a, 18e	In overweight study participants, both mean BMI and WC were significantly increased in MEP Q2 (median, 131 µg/g cr) [21.7 (20.7, 22.8) and 73.5 (70.7, 76.4) respectively], Q3 (235 µg/g cr) [23.8 (22.7, 24.8) and 79.2 (76.3, 82.0) respectively] and Q4 (948 µg/g cr) [23.5 (22.5, 24.3) and 78.8 (76.3, 81.3), respectively], compared to MEP Q1 (67 µg/g cr). Similar relationships were also found for LMWP. Null associations of other phthalate metabolites with anthropometric measures of obesity were observed among the children.
(111)	Cross-sectional (USA) Children, adolescents and adults (4369)	MBP, MiBP, MEP, MEHP, MCP, MNP, MOP, MBzP	6–80 y	6–80 y	1, 4, 5b, 6h, 7, 9df, 10a, 16, 18b	In male (20–59 y) groups, both BMI and WC increased from Q1 to Q4 of MBzP (mean BMI=26.7, 27.2, 28.4, 29.0, *p*=0.0002). Mean BMI generally decreased across quartiles among adolescent girls (25.1, 23.7, 23.0, and 23.6 from the Q1 to Q4 of MEHP, *p-trend*=0.02) and among 20–59 y females (29.8, 30.2, 28.5, and 28.1, *p-trend*=0.02). Most of the coefficients for MEP were positive, with the exception of adolescent males (no relationship) and older females (an inverse relationship). For adolescent girls, mean BMI were 22.9, 23.8, 24.1, and 24.7 (*p-trend*=0.03) with increasing quartile of metabolite concentration, and adjusted mean WC were 77.4, 79.7, 80.1, and 81.6 (*p-trend*=0.02).
(112)	Cross-sectional (USA), Male (1451)	MBP, MEP, MEHP, MEHHP, MEOHP, MBzP	>18 y	>18 y	1, 6f, 7b, 9a, 10a, 18b	Ln-transformed MBzP, MEHHP, MEOHP, and MEP concentrations were positively associated with WC [β=1.29 (SE: 0.34), β=1.71 (SE: 0.56), β=1.81 (SE: 0.60) and β=0.77 (SE: 0.29), respectively] after adjusted with covariates (*p ≤* 0.013).

n, number; y, year; m, month; T_r_, trimester; T, tercile/tertile; Q, quartile/quantile; cr, creatinine; GO, general obesity; AO, abdominal/central obesity; PA, phthalic acid; MBP, mono-butyl phthalate/mono-n-butyl phthalate; MBzP, mono-benzyl phthalate; MiBP, mono-isobutyl phthalate; MMP, mono-methyl phthalate; MEP, mono-ethyl phthalate; MEHP, mono-2-ethylhexyl phthalate; MEOHP, mono (2-ethyl-5-exohexyl) phthalate; MEHHP, mono (2-ethyl-5hydroxyhexyl) phthalate; MEHP, mono-ethylhexyl-phthalate; MECPP, mono-(2-ethyl-5-carboxypentyl) phthalate; DBP, dibutyl phthalate; DEP, diethyl phthalate; MCNP, mono-(carboxylnonyl) phthalate; MCOP, mono-(carboxyoctyl) phthalate; MCMHP, mono-2-carboxymethyl-hexyl phthalate; MNP/MiNP, mono-isononyl phthalate; MCP, monocyclohexyl phthalate; MCPP, mono-(3-carboxypropyl) phthalate; MOP/MnOP, mono-n-octyl phthalate; MHxP, monohexylphthalate; MHpP, mono-2-heptylphthalate; MHBP, mono(4-hydroxybutyl) phthalate; MCHP, monocyclohexyl phthalate; 7OHMMeOP, mono(4-methyl-7-hydroxyoctyl) phthalate; LMWP, low molecular weight phthalate; HMWP, high molecular weight phthalate; SDS, standard deviation score; BMI, body mass index; BMIZ, BMI z-score; BSA, body surface area; WC, waist circumference; WCZ, WC z-score; ST, skinfold thickness; HC, hip circumference; WHtR, weight to height ratio; BF, body fat; FM, fat mass; PCA, principle component analysis.

^1^age (^a^child age, ^b^maternal age, ^c^age squared); ^2^sex (^a^child sex); ^3^education level (^a^maternal/paternal education); ^4^socioeconomic status (^a^household/family income, ^b^poverty to income ratio, ^c^insurance, ^d^maternal receipt of public assistance); ^5^physique (^a^pre-pregnancy/maternal BMI, ^b^height, ^c^gestational weight gain, ^d^birth weight); ^6^food (^a^maternal diet quality score, ^b^total energy/calorie intake, ^c^child`s food insecurity and fast-food consumptions at each point, ^d^food security, prenatal fruit/vegetables and fish consumptions, ^e^prenatal vitamin use, ^f^total fat intake, ^g^breast feeding, ^h^dietary factors); ^7^smoking (^a^smoking during pregnancy, ^b^serum/urinary cotinine); ^8^alcohol consumption/drinking status; ^9^activity (^a^physical activity, ^b^exercise, ^c^recreational activity, ^d^TV/video watching time, ^e^sedentary hours, ^f^metabolic equivalent hours); ^10^race (^a^maternal/paternal/child race/ethnicity, ^b^maternal country of origin); ^11^information of pregnancy (^a^parity, ^b^gestational age, ^c^cesarean section and delivery experience); ^12^maternal marital status; ^13^location of participants or study (^a^site/area, ^b^housing type, ^c^years in US prior to delivery, ^d^cohort); ^14^past history (^a^lipid profile, ^b^SBP/DBP, ^c^blood sugar, ^d^depressive symptoms, ^e^diabetes) ^15^job/occupation (^a^work status during pregnancy); ^16^reproductive factors (^a^tanner stages, ^b^puberty onset, ^c^menopausal status/hormone use); ^17^using cosmetics, plastic packaging and bottled drinks; ^18^others (^a^urinary specific gravity, ^b^urinary creatinine level, ^c^phthalic acid, ^d^prenatal BPA, ^e^urine dilution and collection date, ^f^an interaction term between age and phthalate concentration categories, ^g^an interaction term between age squared and phthalate concentration categories, ^h^an interaction term between race/ethnicity and age).

[In all cases in the outcome, ranges within the first bracket indicate the 95% CI.]

Associations between maternal 1^st^ trimester DEHP exposure levels and obesity measures in preschoolers, school-aged children, and adolescents were inconsistent (50, 70, 87, 93). Similarly, the individual or sum of maternal 2^nd^ trimester urinary DEHP metabolites showed both positive and null associations with different obesity indices in preschoolers, school-aged children, and adolescents (50, 87, 95, 96). However, negative or null associations were found between the 2^nd^ and 3^rd^ trimester DEHP exposure levels and anthropometric obesity indices in infants, preschoolers, school-aged children, and adolescents (50, 87, 98–101). Infant (1 year) exposure to DEHP was negatively associated with obesity indices at 8 years of age. In contrast, preschoolers exposed to DEHP (4 and 5 years) were negatively or positively associated (93, 96). Associations of DEHP exposure levels at 6–19 years of age (individual metabolite levels or sum of levels) with overweight or obesity indices in school-aged children and adolescents were very inconsistent (89–92, 94, 96, 102, 105–111). Most of the studies that recruited adults and elderly people reported positive or null associations between one or more DEHP metabolites or the sum of DEHP and different overweight and obesity indices in overall adult and elderly populations or after sex stratification (2, 48, 49, 65, 88, 97, 104, 106, 111, 112).

Similar inconsistent associations were also found among non-DEHP metabolites [mono-butyl phthalate (MBP), mono-ethyl phthalate (MEP), mono-methyl phthalate (MMP), mono-benzyl phthalate (MBzP), mono-isobutyl phthalate (MiBP), mono-(carboxylnonyl) phthalate (MCNP), mono-isononyl phthalate (MINP), and others], and obesity indices at different stages of life. First to 3^rd^-trimester maternal urinary concentrations of non-DEHP metabolites (except MCPP) displayed null or negative associations with anthropometric parameters of obesity in preschoolers, school-aged children, and adolescents (50, 70, 93, 98, 99, 101, 103). In contrast, one study found positive associations between maternal urinary concentrations of MEP, MBP, MBzP, and MiBP and obesity indices among all study participants (95). Another study also found positive associations after sex-stratified analysis in both males (MBzP) and females (MiBP and MBP) (87). Exposure levels of non-DEHP metabolites in toddlers and preschoolers showed null associations with their obesity measures (96, 101). However, one study described positive associations between MEP, MBP, and MBP, and obesity indices in girls, with negative associations in boys (93). Exposure levels of school-aged to adolescents to non-DEHP metabolites (MMP, MEP, MBP, MiBP, and MBzP) were mostly positively associated with one or more anthropometric indices in school-aged children or adolescents (89, 91, 92, 94, 96, 102, 105–110). In contrast, after sex stratification, inconsistent associations were evident (94, 102, 107, 110). One study recruited subjects 6–80 years old and found inconsistent associations among non-DEHP metabolite concentrations at different exposures (6-11, 12-19, 20-59 and 60-80 y) and corresponding outcome assessment ages (111). Exposure levels of non-DEHP metabolites in adults and the elderly also showed null or positive associations with their overweight and obesity indices (2, 48, 49, 65, 88, 97, 104, 106, 112). One study evaluated ethnicity-dependent association and found that higher maternal urinary concentrations of MCPP heightened the odds of being overweight or obese in Hispanics than in non-Hispanic blacks, although null associations were found with BMI (99). Prepubertal girls showed positive associations between %MEHHP and BMI, WC, and %BF, and showed significant odds increase in the 3^rd^ and 4^th^ quartiles compared to the 1^st^ quartile. The relationship was null in pubertal girls (90).

Data regarding the lowest threshold levels of phthalate metabolites for overweight or obesity outcomes in humans are limited. Low molecular weight phthalate (LMWP) metabolite concentrations ≥0.27 µmol/ml were associated with significantly increased overweight or obesity indices in male children and adolescents (106). Another study reported increased BMI and WC for median urinary MEP concentrations ≥131 and ≥948 µg/g creatinine, respectively (110).

### Environmental POPs and Obesity

A total of 41 human epidemiological studies (33 cohort and 8 cross-sectional studies) explored the relationships between *in utero* and early life exposure to POPs and anthropometric indices of overweight and obesity among infants, children, adults, and elderly populations (**Table 3**). The studies assessed POP levels in blood (serum/plasma) or umbilical cord blood (whole blood, serum/plasma).

**Table 3:** Associations of environmental persistent organic pollutants with anthropometric overweight and obesity indices.

Ref.	Study type (country), Subjects (n)	Exposure		Outcome ass. time	Covariate	Key findings
		Markers	Biomonitoring time			
(4)	Birth cohort (China), Mother-child (318)	9 PBDEs	At birth (cord serum)	7 y	1ab, 2a, 3a, 4a, 5a, 7a, 9a	Cord serum BDE153 and BDE154 concentrations were significantly associated with lower childhood BMIZ [β=−0.15 (−0.29, −0.02) and β=−0.23 (−0.43, −0.03)], respectively and lower WC [β=−0.75 (−1.43, −0.06) and β=−1.22 (−2.23, −0.21)], respectively. Prenatal BDE154 exposure was related to decreased risk of obesity for children aged 7 y [OR=0.46 (0.22, 0.94)]. On the other hand, BDE153 and BDE154 showed significant negative associations with BMIZ, WC, and obesity only in boys.
(51)	Birth cohort (Denmark), Mother-child (649)	PFHxS, PFOS, PFOA, PFNA, PFDA	11.3 GW (median)	3 m, 18 m	1a, 2, 3a, 5a, 7a, 11a, 17a	At 3 m and 18 m of age, 1 ng/ml increases in PFOA, PFNA, and PFDA were associated with average increases in the PI SDS of 0.07 (0.01, 0.13), 0.24 (0.08, 0.41), and 0.60 (0.18, 1.02), respectively and BMI SDS of 0.18 (0.02, 0.34), 0.42 (0.01, 0.84), and 0.04 (−0.01, 0.10), respectively. In girls aged 3 m and 18 m, PFNA and PFDA concentrations were associated with increased BMI SDS [PFNA: 0.26 (0.03, 0.49), PFDA: 0.58 (−0.03, 1.19)] and PI SDS [PFNA: 0.36 (0.13, 0.59), PFDA: 1.02 (0.40, 1.64)]. Associations were null in boys. PFNA and PFDA were positively associated with %BF SDS at 3 m [β=0.20 (0.06, 0.34)] and [β=0.40 (0.04, 0.75)] for 1 ng/ml increases, respectively), but not at 18 m.
(3)	Birth cohort (USA), Mother-child (212)	PFOS, PFOA, PFNA, PFHxS	∼16 GW, at birth, 3, 8, 12 y	12 y	1ab, 2a, 3a, 5a, 7a, 10a, 11a, 12, 16	Serum PFOA and PFHxS concentrations during pregnancy were associated with increase in AO across all anthropometric measures and overweight/obesity. A 2-fold increase in prenatal PFOA concentration was associated with WtHR [β=0.02 (0.00, 0.03)] but not with WC [β=2.0 (−0.8, 4.8)] and other obesity indices. PFOA and PFHxS concentrations during pregnancy were associated with higher overall obesity and AO across all measures in girls, while non consistent results found in boys. Childhood PFAS concentrations were not associated with adiposity measures.
(113)	Birth cohort (China), Mother-child (404)	PFOS, PFOA, PFNA, PFDA, PFUA, PFBS, PFDoA, PFHxS	At birth (cord blood)	5 y	1a, 3a, 5a, 7b, 11a, 17a	In girls, a 10-fold increase in PFBS concentration was associated with increases in WC [β=1.48 (0.32, 2.53)], FM [β=0.50 (0.008, 0.99)], %BF [β=1.79% (0.04, 3.54)], and WHtR [β=0.01 (0.001, 0.02)]. Girls at T3 of PFBS concentrations had higher WC [β=2.06 (0.43, 3.68)], FM [β=0.79 (0.08, 1.51)], %BF [β=2.84 (0.29, 5.39)] and WHtR [β=0.01 (0.0008, 0.03)] in compared with T1. Increased PFDoA concentrations were associated with lower WC [β=−1.95 (−3.61, −0.3)], FM [β=−0.93 (−1.65, −0.2)], and %BF [β=−3.02 (−5.61, −0.43)] at T2 compared with T1 girls. PFNA concentrations were associated with higher %BF [T3 *vs* T1: β=2.16 (0.07, 4.25)] in boys. Other PFAS showed null associations with obesity indices.
(114)	Cohort (USA), Children (206)	PBDEs (BDE 28, 47, 99, 100, 153)	1, 2, 3, 5, 8 y	8 y	1ab, 2a, 4a, 5a, 6ab, 7a, 8, 9abc, 10a, 12, 14a	A 10-fold increase in BDE153 concentration at 1, 2, 3, 5, and 8 y were associated with lower %BF of 2.0% (−3.9, −0.1), 2.9% (−4.9, −0.9), 3.6% (−5.5, −1.7), 5.6% (−7.8, −3.4), and 6.9% (−9.1, −4.7), respectively. Associations were stronger in boys. A 10-fold increase in BDE153 concentration at 2, 5, and 8 y were associated with a decrease of 4.0 cm (−6.9, −1.1), 7.3 cm (−10.5, −4.0), and 9.3 cm (−12.5, −6.1) in WC.
(115)	Birth cohort (Norway and Sweden), Mother-child (412)	PFOS, PFOA, 7 PCBs, HCB, p, p′-DDE, oxychlordane, p, p′-DDT, β-HCH, t-NC	<20 GW	5 y	1a, 3a, 5ab, 6c, 7a, 10b, 11a	A 2.72-fold increase in maternal serum PFOS concentrations was associated with increased BMIZ [β=0.18 (0.01, 0.35)] and triceps ST z-score [β=0.15 (0.02, 0.27)] in children. Overall, a 2.72-fold increase in maternal serum PFOS and PFOA concentrations were associated with increased odds for child overweight/obesity [OR=2.04 (1.11, 3.74) and OR=1.61 (0.75, 3.46), respectively]. But greater odds were reported among Norwegian children [OR=2.96 (1.42, 6.15)] for PFOS and [OR=2.90 (1.10, 7.63) for PFOA. PFOS and PCB153 concentrations in Swedish children showed dose-dependent associations with child overweight/obesity.
(116)	Birth cohort (South Africa), Mother-child (708)	OCs (p, p′-DDE, p, p′-DDT)	Near delivery	≤2 y	1a, 3a, 4, 5a, 11a, 14bc	Among girls, maternal p, p′-DDT level was associated with higher BMI-for-age [β=0.22 (0.10, 0.35)], weight-for-height [β=0.22 (0.09, 0.34)], and weight-for-age [β=0.17 (0.05, 0.29)]. p, p′-DDE also showed similar associations in a single pollutant model, but not in a Bayesian kernel machine regression model.
(117)	Birth cohort (USA), Women (468)	9 OCs, 10 PBDEs, 35 PCBs	≥18 y	≥18 y	1, 4a, 13a	Positive associations with BMI were found in Q4 *vs* Q1 for p, p′-DDT [β=3.2 (1.5, 4.9)], β-HCH [β=3.6 (2.0, 5.2)] and BDE47 [β=1.9 (0.3, 3.5)], while BDE153 was inversely associated [β=−2.8 (−4.4, −1.2)]. Positive associations were also found for p, p′-DDT, β-HCH, BDE47, PCB74, and PCB99, and inverse associations were found for BDE153 and PCB180 with WC. A significant increasing trend in risk of obesity in Q2, Q3, and Q4 for p, p′-DDT [respectively, RR=1.38 (1.08, 1.76), RR=1.45 (1.13, 1.85), and RR=1.48 (1.16, 1.89)], and β-HCH [respectively, RR=0.99 (0.77, 1.27), RR=1.43 (1.11, 1.84), and RR=1.37 (1.06, 1.77)] were observed. Associations were positive for BDE47 [RR=1.29 (1.03, 1.60)], but were inverse for BDE153 [Q2 *vs* Q4: RR=0.70 (0.56, 0.88)]. PCB99 concentrations were associated with increased risk of obesity (*p*<0.01), while a decreasing trend was observed for PCB180 (*p*=0.03).
(118)	Birth cohort (USA), Mother-child (240)	OCs (o, p′-DDT, p, p′-DDT, p, p′-DDE)	26 GW	12 y	4,5a, 13a	In boys, 10-fold increase in prenatal o, p′-DDT, p, p′-DDT and p, p′-DDE concentrations were associated with increased BMIZ and WCZ [β=0.37 (0.08, 0.65) and β=0.31 (0.07, 0.56)]; [β=0.26 (0.03, 0.48) and β=0.25 (0.05, 0.45)], and [β=0.31 (0.02, 0.59) and β=0.27 (0.01, 0.53)], respectively. Similarly, a 10-fold increase in o, p′-DDT and p, p′-DDT were associated with increased risk of obesity [RR=1.46 (1.07, 1.97)] and [RR=1.28 (1.01, 1.64)], respectively.
(119)	Cross-sectional (Spain), Adults (429)	30 POPs (includes PCBs, DDTs, DDEs)	≥18 y	≥18 y	NC	Median level of p,p′-DDE among participants with BMI ≤25 was significantly lower than that of participants with BMI ≥25 (0.83 μg/l *vs*. 1.26 μg/l, *p*<0.0001). p,p′-DDE identified as a risk factor for the development of overweight [BMI ≥25: Exo (B)=1.38 (1.15, 1.64)], and obesity [BMI ≥30: Exo (B)=1.22 (1.08, 1.38)].
(120)	Birth Cohort (USA), Mother-child (1006 & 876)	PFOA, PFOS, PFHxS, PFNA	<22 GW	3.2, 7.7 y (mean)	1ab, 2a, 3a, 4a, 10a, 11a, 17c	In girls in mid-childhood (7.7 y), each IQR increment of prenatal PFOA concentrations was associated with 0.21 kg/m^2^ higher BMI (−0.05, 0.48)], 0.76 mm higher sum of subscapular and triceps ST, (−0.17, 1.70)], and 0.17 kg/m^2^ higher total FMI (−0.02, 0.36)] Similar associations were observed for PFOS, PFHxS, and PFNA. Null associations found for early-childhood (3.2 y) PFAS concentrations and adiposity measures in boys and girls.
(121)	Birth cohort (Faroe Islands), Mother-child (444)	HCB, DDE, PFOS, PFOA, PCBs, p, p′- PFHxS, PFNA, PFDA	2 wk of postpartum, 5 y	18 m, 5 y	1a, 2a, 5abc, 6d, 7a, 10b, 11ab	A 10-fold increase in maternal HCB concentrations were associated with increased BMIZ at 18 m [β=0.15 (0.01, 0.30)] and at 5 y [β=0.19 (0.04, 0.34)]. Similar associations were found between PFOS concentrations and BMIZ [β=0.23 (0.04, 0.42)] and overweight risk [RR=1.29 (1.01, 1.64)] at 18 m. Associations were null at 5 y. A, 10-fold increase in maternal PFOA was associated with the risk of being overweight at 5 y [RR=1.50 (1.01, 2.24)]. Child serum-POPs (except PFHxS and DDE) levels inversely associated with BMIZ or overweight risk at 5 y.
(122)	Birth cohort (USA), Mother-child (285)	PFOA, PFOS, PFNA, PFHxS	16 GW, 26 GW (mean) and at birth	2−8 y	1a, 3a, 4a, 6ae, 7c, 10a, 11a, 12, 14a, 15a	WC was higher among children in the T2 (4.3–6.4 ng/ml) [β=4.3 (1.7, 6.9)] and T3 (6.6–25 ng/ml) [β=2.2 (−0.5, 4.9)] compared with T1 (0.5–4.2 ng/ml) of prenatal PFOA. Between 2 and 8 y, BMIZ increased at a greater rate among children at T2 [β=0.44 (0.23, 0.64)] and T3 [β=0.37 (0.14, 0.60)] of PFOA compared with T1 [β=0.12 (−0.08, 0.32)]. Children born to women with T2 and T3 PFOA concentrations had increased risk of overweight [RR=1.84 (0.97, 3.50)] or obesity [RR=1.54 (0.77, 3.07)] at 8 y compared to children born to women in the T1 category.
(123)	Birth cohort (USA), Mother-child (318)	10 PBDEs (∑PBDEs: BDEs 28, 47, 99, 100, 153)	16 GW (mean)	1−8 y	1a, 3a, 4a, 7a, 10a, 14a, 6d	Ten-fold increases in BDE100 and ∑PBDEs were associated with decreased WC [β=−1.50 (−2.93, −0.08) and β=−1.57 (−3.11, −0.02), respectively] among children 4–8 y in age. In contrast, a 10-fold increase in BDE153 was associated with lower BMIZ [β=−0.36 (−0.60, −0.13)] at 2–8 y and lower %BF [β=−2.37 (−4.21, −0.53)] at 8 y.
(103)	Birth cohort (Spain), Mother-child (470)	27 POPs including 6 OCs, 6 PBDEs	1^st^, 3^rd^ T_r_	7 y	1ab, 2a,4,5abc, 6c, 7a, 10b, 11c	Maternal concentrations of HCB, βHCH, PCB138, and PCB180 were associated with increased child BMIZ, HCB, βHCH, PCB138, and DDE with overweight risk. In principle component analysis, the OC factors (DDE, HCB, βHCH, PCB138, PCB153, PCB180) were positively associated with BMIZ [T3 *vs*. T1, β=0.37 (0.03, 0.72)] and with overweight [T3 *vs*. T1, RR=2.59 (1.19, 5.63)].
(124)	Birth cohort (Greenland, Ukraine), Mother-child (1022)	PFOA, PFOS	24 GW (mean)	5−9 y	1ab, 2a, 3a, 5a, 7a, 11a	A 2.72-fold increase in pregnancy PFOA concentrations were associated with increased risk of offspring overweight [RR=1.11 (0.82, 1.53)] in Greenlandic children and [RR=1.02 (0.72, 1.44) in Ukrainian children. A 2.72-fold increase of prenatal PFOA and PFOS were associated with increased risk of having WHtR >0.5 [RR=1.30 (0.97, 1.74)] and [RR=1.38 (1.05, 1.82)], respectively, in the total study subjects.
(125)	Cross-sectional (Denmark), Children (509)	PCBs, p, p′-DDE, HCBs	8−10 y	14–16, 20−22 y	3a, 4, 5ad, 6c, 7a	Child ΣPCB concentrations were inversely associated with WC and %BF in girls 14–16 y old (*p*=0.04 and *p*=0.03, respectively). The inverse association between ΣPCB (PCB138, 153, and 180) and BMIZ was evident among those in the T3 (>0.28 μg/g lipid) compared with the T1 (<0.16 μg/g lipid) among women 20–22 y old [β =−0.44 (−0.80, −0.08)].
(126)	Birth cohort (Greece), Mother-child (689)	PCBs, DDE, HCBs	1^st^ T_r_	4 y	1ab, 2a, 3a, 5ac, 6c, 7a, 11ac, 14b	A 10-fold increase in maternal HCB concentrations was associated with a higher BMIZ [β=0.49 (0.12, 0.86)], obesity [RR=8.14 (1.85, 35.81)], AO [RR=3.49 (1.08, 11.28)], and greater sum of ST [β=7.71 (2.04, 13.39)] at 4 y of age. Prenatal DDE exposure was also associated with higher BMIZ [β=0.27 (0.04, 0.5)], AO [RR=3.76 (1.70, 8.30)].
(127)	Birth cohort (Canada), Mother-child (224 & 216)	10 PBDEs, (penta PBDE= BDEs 47, 99, 100, 153, Σ4 PBDE)	26.7 GW (mean), 7 y	7 y	1a, 2, 3a, 4, 5ab, 6df, 11c, 13a	Maternal serum Σ4PBDE concentration was not associated with the BMIZ [β=−0.08 (−0.41, 0.25)], WCZ [β=−0.02 (−2.45, 0.28)], or the odds of being overweight [OR=0.82 (0.38, 1.79)] at 7 y of age. A 10-fold increase in Σ4PBDE concentration was associated with decrease BMIZ in girls [β=−0.41 (−0.87, −0.05) compared with boys [β=0.26 (−0.19, 0.72)]. Child’s serum BDE153 concentration showed inverse associations with BMIZ [β=−1.15 (−1.53, −0.77)] and WCZ [β=−0.95 (−1.26, −0.64)] at 7 y of age.
(128)	Cross-sectional (USA), Adults (2358)	POPs (β-HCH, dioxins, PCBs and few others)	≥20 y	≥20 y	1, 2, 3, 7d, 8, 9d, 10a, 11a, 13b	β-HCH, heptachlorodibenzo-p-dioxin, octachlorodibenzo-p-dioxin, and PCB126 showed stronger positive correlations, whereas PCBs with ≥6 chlorines inversely correlated with trunk %FM than with leg %FM. Stronger inverse correlations existed between POPs and trunk %FM mainly in participants <40 y of age. Stronger positive correlations between POPs and trunk %FM were observed in older participants.
(129)	Cross-sectional (Belgium), Child (114)	PCBs, dioxins, p, p′-DDE -DDE, HCB	At delivery	7−9 y	1ab, 5a, 3a, 7a,	In unadjusted analysis, prenatal exposure to HCB and p,p′-DDE were significantly and positively associated with BMIZ, WC, WHtR, and sum of four ST. After adjustment, a 2.72-fold increase in prenatal p,p′-DDE concentrations were associated with WC [β=1.02 (1.00, 1.03)] and WHtR [β=1.04 (1.01, 1.07)] in girls.
(130)	Birth cohort (Faroe Islands), Mother-child (656)	PCBs, DDE	34 GW	5 y, 7 y	1a, 11a	The Q4 (>1.95 µg/g lipid) of prenatal PCB exposure was associated with increased BMI [β=2.07(0.59, 3.55)] at age 7 y in girls with overweight mothers. High prenatal PCB and DDE exposure was associated with increased BMI [β=1.2 (0.42, 2.05) and β=1.11 (0.30, 1.92), respectively] and WC [β=2.48 (1.10, 3.85) and β=2.21 (0.84, 3.56), respectively] from 5 to 7 y of age. PCB was associated with increased WC in girls both with overweight (β=2.48) and normal-weight mothers (β=1.25), whereas DDE was associated with increased WC only in girls with overweight mothers (β=2.21).
(131)	Cross-sectional (Belgium), Adults (151)	28 PCBs, p, p′-DDE	≥18 y	≥18 y	NC	Log_10_-transformed serum PCBs levels, but not p, p′- DDE, showed an inverse relationship with weight and BMI in spearman correlation analysis (*p*<0.01). Total serum POPs levels (sum of 28 PCBs and p, p′-DDE) were positively associated with WC and WtHR (*p*<0.01).
(132)	Birth cohort (Spain) Mother-child (1198)	DDT, DDE, HCB, β-HCH, PCBs (153, 138, 180)	1^st^ T_r_	14 m	1ab, 2a, 3a, 5a, 6c, 7a, 10b, 13c	A 10-fold increase in DDE and HCB were associated with overweight [RR=1.15 (1.03, 1.28) and RR=1.19 (1.05–1.34), respectively]. Effect of 10-fold increase in DDE on overweight was stronger in infants who were breastfed ≤16 weeks compared with those breastfed for a longer period [RR=1.26 (1.11, 1.43) and RR=1.02 (0.86, 1.21), respectively].
(133)	Birth cohort (Greenland, Poland, Ukraine), Mother-child (1109)	PCB153, p,p′-DDE	9–40 GW, 12 m	5−9 y	1a, 3a, 5ae, 6c, 7a, 8, 9d, 11a, 17a	Null associations were found between pregnancy PCB153 and p, p′-DDE, and child BMI in T3 *vs* T1 were [β=−0.07 (−0.32, 0.18)] and [β=−0.10 (−0.30, 0.10)], respectively. Null associations were also observed for estimated postnatal PCB153 and p, p′-DDE concentrations during the first 12 m of life and BMI in T3 *vs* T1 [β=0.12 (−0.15, 0.39)] and [β=−0.03 (−0.20, 0.27)], respectively, at 5–9 y.
(134)	Birth cohort (USA), Mother-child (261)	o, p′-DDT, p, p′-DDT, p, p′-DDE	26 GW	9 y	5a, 13a	Among boys, 10-fold increases in lipid adjusted prenatal DDT and DDE concentrations were associated with increased odds of being overweight or obese [o, p′-DDT: OR=2.5 (1.0, 6.3); p, p′-DDT: OR=2.1 (1.0, 4.5); and p, p′-DDE: OR=1.97 (0.94, 4.13)]. Similar results were found for increased WC and o, p′-DDT [OR=1.98 (0.95, 4.11)], p, p′-DDT [OR=2.05 (1.10, 3.82)] and p, p′-DDE [OR=1.98 (0.97, 4.04). Positive associations were also observed among prenatal exposure levels of DDT and DDE metabolites with BMIZ, WCZ and %BF.
(135)	Birth cohort (USA), Mother-child (270)	DDT, DDE	≈26 GW	2−7 y	5ac, 9b,	A 10-fold increase in o, p′-DDT, p, p´-DDT and p, p′-DDE was associated with obesity [o, p′-DDT, OR=1.17 (0.75, 1.82); p, p′-DDT, OR=1.19 (0.81, 1.74); p, p′-DDE, OR=1.22 (0.72, 2.06)], and BMIZ [p, p′­DDE, β=0.12 (−0.11, 0.35)]. Significant positive associations were found between DDT and DDE exposure levels with increasing age (2, 3.5, 5, and 7 y) and obesity.
(136)	Birth cohort (USA), Mother-child (1915)	11 PCBs, β-HCH, DDT, HCB, t-NC dieldrin	3^rd^ T_r_	7 y	1b, 3a, 4, 5a, 7a, 10a, 11d, 13c,14b	Null associations were found between exposure to OCs and BMI, and overweight/obesity after adjustment of potential covariates. Dieldren was associated with obesity [Q4 (0.92–1.18 μg/l) *vs* Q1(< 0.57 μg/l), OR=3.6 (1.3–10.5) and Q5 (>1.18 μg/l) *vs* Q1, OR=2.3 (0.8–7.1)].
(137)	Birth cohort (Denmark), Mother-child (1400)	PFOA, PFOS	1^st^-2^nd^ T_r_	7 y	1ab, 4, 5a, 7a, 10a, 11a, 17a	Maternal PFOS (7.3 to ≤44 ng/ml for boys and 6.4 to ≤43.5 ng/ml for girls) and PFOA (0.5 to ≤7.10 ng/ml for boys and 1.10 to ≤6.70 ng/ml for girls) concentrations were not associated with BMI or overweight at 7 y.
(138)	Birth cohort (Denmark), Mother-child (665)	PFOA, PFAS, PFOSA, PFNA	30 GW	20 y	1ac, 3a, 5ac, 7a, 11a	Maternal PFOA concentration was associated with GO and AO at female offspring [Q4 *vs* Q1 (median: 5.8 *vs*. 2.3 ng/ml): RR=3.1 (1.4, 6.9) and RR=3.0 (1.3, 6.8), respectively]. Maternal PFOS, PFOSA and PFNA concentrations were not associated with offspring BMI and WC.
(139)	Birth cohort (Spain), Child (344)	HCB, DDE, DDT, PCBs	At birth (Cord blood)	6.5 y	1a, 3a, 5ac, 6b, 7a, 11a, 15b	Increased risk of overweight was observed in the T3 of cord blood PCB concentrations [T3 (>0.9 ng/ml) *vs* T1 (<0.6 ng/ml), RR=1.70 (1.09, 2.64)] and the T2 of DDE exposure [T2 (0.7–1.5 ng/ml) *vs* T1(<0.7 ng/ml), RR=1.67 (1.10, 2.55)], but null association with DDT exposure in overall population. A significant association was found for PCBs and overweight in the T3 *vs* T1 in girls [RR=2.13 (0.99, 4.57)] than in boys [RR=1.43 (0.82, 2.48)]. Similar associations also found for DDE.
(140)	Cross-sectional (USA), Women (109)	36 PCBs, 9 OCs,	50−75 y	50−75 y	1, 3, 4a, 5c, 11e, 12	Plasma PCB180 concentrations were negatively and significantly associated with BMI, %BF, subcutaneous fat, intra-abdominal fat, WC, hip circumference, and WtHR. PCB118 showed significant positive associations with subcutaneous fat, intra-abdominal fat, WC, and WtHR. Conversely, PCB105 and p, p′-DDE were generally increased or showed null association with these obesity indices.
(141)	Cohort (Sweden), Elder people (970 & 511)	16 PCBs, 3 OCs, BDE, dioxin	70, 75 y	70, 75 y	6g, 7d, 8, 14b, 9e	In the cross-sectional analyses, concentrations of the less chlorinated PCBs, p, p′- DDE and dioxin had adjusted odds ratios of 2 to 3 for AO. Highly chlorinated PCBs were inversely associated. In the prospective analyses, similar but slightly weaker associations were seen between POPs and risk of development of abdominal obesity.
(142)	Birth cohort (Spain), Mother-child (657)	HCB, 2,2 DDE, 2,2 DDT, β-HCH, 4 PCBs	1^st^ T_r_	14 m	1ab, 5fg, 7a, 10b,	A 10-fold increase in prenatal serum DDE was associated with elevated BMIZ [RR=1.50 (1.11, 2.03) for normal pre-pregnancy-weight mothers, and RR=1.40 (1.12, 1.75) for all mothers] at 14 m. OCs were positively associated with rapid weight gain and subsequent development of overweight.
(143)	Cohort (African and white American), Adults (90)	8 OCs, 22 PCBs, PBB	18−30 y	18−30 y	1, 2, 5d, 10a, 14b	Among OCs, p, p′-DDE predicted higher BMI forming inverted U-shaped dose-response relations at 20 y after adjusting for the baseline values (*p*_quadratic_<0.01, from Q1 to Q4). Persistent PCBs with ≥7 chlorides predicted higher BMI at 20 y with similar dose-response curves.
(144)	Cross-sectional (Belgium), Adults (144)	4 PCBs, p, p′-DDE, β-HCH	≥18 y	≥18 y	NC	A significant negative correlation between serum levels of PCB153, 180, 170 and sumPCBs, and BMI, WC, and %FM in entire groups (lean and obese together). Conversely, β-HCH showed significant positive correlation with WtHR, BMI, WC, and %FM in entire groups.
(145)	Cross-sectional (Sweden), Elder people (890)	16 PCBs, 3 OCs, BDE47, dioxin	70 y	70 y	2, 3, 5hi, 6g, 7d, 8, 9d	Plasma concentrations of the PCB105, PCB118 and HCB, TNK, and DDE were all positively related to FM (*p ≤* 0.03). Subjects in the Q5 for PCB105 and PCB118 showed a mean FM that was 4.8 (3.0, 6.7) and 4.6 (2.8, 6.5) more than subjects in the Q1. In contrast, the PCB156, 157, 169, 170, 180, 189, 194, 206, and 209 were negatively related to FM (*p*=0.0001). For PCB194, subjects in the Q5 showed a mean FM that was 10.8 less than subjects in the Q1.
(146)	Birth cohort (Denmark) Mother-child (1400)	PFOA, PFOS	1^st^−2^nd^ T_r_	5 m, 12 m	1a, 4, 5a, 6c, 7a, 11a, 17a,	A 1 ng/ml increase in the maternal blood levels of PFOS were inversely associated to children’s weight, after adjustment [β=−5.8 (−10.4, −1.2)] at 12 m. Maternal PFOA concentrations was also associated with BMIZ at 12 m of age [β=−0.007 (−0.011, −0.002)].
(147)	Birth cohort (USA), Mother-child (151&129)	PCBs, DDE	During pregnancy	20−50 y	1b, 5ach, 6b, 11f, 13b, 17d	Compared with maternal DDE levels of <1.503 µg/l, daughter weight was 5.93 g higher when prenatal DDE levels were 1.503–2.9 µg/l, and 9.92 g if levels were >2.9 µg/l, and offspring BMI was 1.65 times higher when prenatal DDE levels were 1.503–2.9 µg/l and 2.88 if levels were >2.9 µg/l. Prenatal PCBs showed null associations with offspring weight and BMI.
(148)	Birth cohort (Belgium), Mother-infant (138)	5 PCBs, HCB, DDE	At birth (Cord blood)	1−3 y	NC	Increasing concentrations of cord blood PCBs were associated with higher BMI SDS values at 1–3 y of ages [β=0.003(0.001); *p*=0.03]. p, p′-DDE had a small effect on BMI SDS in children of nonsmoking mothers but smoking enhanced the relation between DDE and BMI SDS at 3 y.
(149)	Birth cohort (Spain), Children (482)	HCB, 7 PCBs, p, p′-DDE, p, p′-DDT	At birth (Cord blood)	6.5 y	1ab, 2, 3a, 5cj, 7a, 11a	Children with HCB levels >1.03 ng/ml in cord blood had a higher BMI [β=0.80 (SE:0.34)] than children with HCB levels < 0.46 ng/ml. Prenatal exposure to HCB was also associated with an increased risk of being overweight [RR=1.69 (1.05, 2.72)] and obese [RR=2.02(1.06, 3.85)] at 6.5 y. A 10-fold increase in HCB concentrations at birth associated with reduced BMI and weight at age 6.5 (β=0.39 and 0.84, respectively), in the children from normoweight women.

n, number; y, year; m, month; T_r_, trimester; T, tercile/tertile; Q, quartile/Quantile; NC, not considered; POPs, persistent organic pollutants; OC, organochlorines; P/BDEs, poly/brominated diphenyl ethers; PFAS, per and polyfluoroalkyl substances; PFOA, perfluorooctanoate; PFOS, perfluorooctane sulfonate; PFOSA, perfluorooctane sulfonamide; PFNA, perfluorononanoate; PFHxS, perfluorohexane sulfonic; PFBS, perfluorobutanesulfonic acid; PFDoA, perfluorododecanoic acid; PFDA, perfluorodecanoic acid; PCB, polychlorinated biphenyl; HCB, hexachlorobenzene; DDE, dichlorodiphenyldichloroethylene; DDT, dichlorodiphenyltrichloroethane; β-HCH, β-hexachlorohexane; t-NC, trans-nonachlor; TNK, transnonachlordane, SDS, standard deviation score; IQR, inter quartile range; GW, weeks of gestation; RR, relative risk; OR, odd ratio; BMI, body mass index; BMIZ, BMI z−score; FM, fat mass; BF, body fat, ST, skinfold thickness; WC, waist circumference; WCZ, WC z−score; WHtR, waist to height ratio; WtHR, weight to height ratio; PI, ponderal index; GO, general obesity; AO, abdominal/central obesity.

^1^age (^a^maternal age/maternal age at delivery, ^b^child age, ^c^offspring age at follow up); ^2^sex (^a^child sex); ^3^education level (^a^maternal/paternal education); ^4^socioeconomic status (^a^household/family income); ^5^physique (^a^pre-pregnancy/maternal BMI, ^b^gestational weight gain, ^c^birth weight, ^d^baseline BMI/obesity, ^e^paternal BMI, ^f^parental overweight/obesity, ^g^rapid growth status, ^h^height/maternal height, ^i^lean mass, ^j^pre-pregnancy obesity); ^6^food (^a^prenatal vitamin use, ^b^breast feeding/breast fed, ^c^duration of breastfeeding, ^d^maternal fish intake during pregnancy, ^e^maternal diet, ^f^child diet, ^g^total energy/calorie intake); ^7^smoking (^a^active/passive smoking during pregnancy, ^b^paternal smoking during pregnancy, ^c^maternal serum cotinine, ^d^cigarette smoking); ^8^alcohol consumption/drinking status; ^9^activity (^a^time playing outside, ^b^TV/video watching time, ^c^time playing video games, ^d^child physical activity/physical activity, ^e^regular exercise); ^10^race (^a^maternal/paternal/child race/ethnicity, ^b^maternal country of origin/birth); ^11^information of pregnancy (^a^parity/interpregnancy interval, ^b^type of delivery, ^c^child gestational age, ^d^child birth order, ^e^number of live births, ^f^numbers of offspring pregnancies); ^12^maternal marital status; ^13^location of participants or study (^a^years of USA residence/time in the US at birth, ^b^history of lactation, ^c^study center/study sub-cohort); ^14^past history (^a^depressive symptoms, ^b^lipid profile, ^c^HIV status) ^15^occupation/job (^a^work status during pregnancy, ^b^social class); ^16^reproductive factors; ^17^others (^a^gestational age at blood drawing, ^b^visit, and interaction between visit and PFAS, ^c^time of blood draw, ^d^prenatal PCBs).

[In all cases in the outcome, ranges within the first bracket indicate the 95% CI].

A total of 8 epidemiological studies (7 cohort and one cross-sectional study) investigated the associations of several PBDEs with anthropometric measures of obesity along with other POPs in children, adults, and elderly individuals. Inconsistent associations were documented (**Table 3** and **Matrix Table 4**). PBDE congeners, including BDE28, BDE47, BDE99, BDE100, BDE153, and BDE154, were mainly associated with obesity indices. In most of the included studies, the BDE153 congener was negatively associated with one or more overweight or obesity indices in children and adults (4, 114, 117, 123, 127). All other PBDE congeners (BDE28, BDE47, BDE99, BDE100, BDE154, BDE209, and sum of PBDE), except BDE154, showed null associations with obesity indices (4, 103, 114, 117, 127, 141, 145). One study instead showed a positive association between BDE47 and BMI in adults ≥18 years of age (117). Associations of PBDE congeners with obesity in elderly people aged ≥70 years were null in two separate studies (141, 145). Early childhood exposure to PBDEs was negatively associated (BDE153) or inconsistent (others) with obesity indices, especially BMI and WC, at 7 years of age (114, 127).

Eleven birth cohort studies investigated the associations between *in utero* or maternal and childhood exposure to PFAS and obesity indices. Associations between maternal exposure levels of PFAS metabolites and obesity indices were inconsistent (**Table 3** and **Matrix Table 5**). First- to 2^nd^-trimester exposure levels of PFOS and PFOA showed inconsistent associations with the obesity indices of infants and toddlers (51, 146). In contrast, almost all studies found positive associations between maternal PFOA concentrations and different obesity indices in overall and/or sex-stratified populations of preschoolers and school-aged children (3, 115, 121, 122, 124), with two exceptions: *in utero* PFOA exposure showed a null association with BMI, WC, and overweight in school-aged children (120, 137). In contrast, PFOS and other PFAS levels were inconsistently associated with anthropometric measures of overweight and obesity in preschoolers and school-aged children (113). Prenatal exposure levels of PFOA were positively associated only in adult females, but the associations were null for all PFASs when considering the overall population (138). However, exposure levels of PFAS in preschoolers and school-aged children mostly showed null or negative associations with overweight or obesity indices (3, 120, 121). Gestational exposure levels of PFOA ≥4.3–6.4 ng/ml were associated with increased WC in the children 2–8 years of age (122). In contrast, 1^st^ and 2^nd^ trimester exposure levels of PFOA (0.5– ≤7.10 ng/ml in boys and 1.10 to ≤6.70 ng/ml in girls) showed null associations with BMI or overweight at 7 years of age (137).

Positive, negative, or null associations have also been reported between *in utero* or prenatal and postnatal, and between early childhood to the elderly concerning exposure to OCs and overweight and obesity indices (**Table 3** and **Matrix Table 6**) (103, 115–119, 121, 125, 126, 128–136, 139–145, 147–149). Maternal 1^st^ to 3^rd^ trimester blood and/or umbilical cord blood levels of OC metabolites, especially DDE and HCB levels, were positively associated with different anthropometric indices of obesity, whereas associations of PCBs, DDT metabolites, and β-HCH concentrations were null or positive in toddlers and preschoolers (115, 116, 126, 132, 142, 148). Inconsistent associations (positive and null) were also found between PCBs, DDT metabolites, DDE, HCB, and β-HCH levels in the 1^st^ to 3^rd^ trimester maternal blood or umbilical cord blood and obesity indices in school-aged children (103, 118, 130, 133–136, 139, 149). One study found positive associations of 2-week postpartum HCB levels, but not other OCs, with anthropometric indices in 18-month-old and 5-year-old children (121). Among the OCs, DDT and its metabolite DDE showed potent positive associations with obesity indices in the overall population or in school-aged boys and girls (103, 118, 130, 134, 135). Only one study investigated the relationship between prenatal exposure levels of DDE and adult obesity measures, and subsequently addressed the positive associations of adults aged 20–50 years. PCBs showed null associations in the same study participants (147). Again, associations sometimes varied among the countries within the study. A prospective cohort study of 412 Norwegian and Swedish mother-child pairs observed a non-monotonic dose-response relationship between PCB-153 concentrations and child overweight/obesity among Swedish children at 5 years of age, but not in Norwegian children (115). Exposure levels of PCB153 and DDE metabolites in infants were not associated with obesity measures in preschool and school-aged children (133). Early childhood or preschooler exposure levels of HCB, DDE, and PCBs were negatively associated with anthropometric parameters in preschoolers (121). School-aged exposure levels of PCBs, DDE, and HCB showed inconsistent associations with obesity indices in school-aged children, adolescents, and adults (125, 129). Exposure levels of DDE and β-HCH in adults (≥18 years) showed positive associations, PCBs showed inconsistent associations (positive and negative), and other OCs also showed null associations with different anthropometric indices of overweight and obesity (117, 119, 128, 131, 143, 144). OC exposure in elderly people also showed contradictory findings. DDE exposure levels showed positive or null associations, whereas PCBs showed very inconsistent associations (positive, negative, and null) with anthropometric indices in elderly people aged 50 to 75 years (140, 141, 145). Furthermore, cord blood HCB levels >1.03 ng/ml were associated with increased BMI in children at 6.5 years of age (149).

## Discussion

### Controversies and Elucidation

We present evidence of the relationship between urinary/blood levels of selected EOs and their metabolites or congeners, and anthropometric overweight and obesity indices. These relationships are contentious. Prenatal or *in utero*, newborn, and early childhood to elder life exposure to selected EOs might contribute to the development of adiposity at different stages of life, although the findings were inconsistent depending on exposure and outcome assessment periods. Some studies have clarified positive associations, whereas other studies described negative or null associations for the same EO exposure levels and the subsequent anthropometric indices of obesity (**Tables 1**–**3**).　

A representative example is two separate birth cohort studies from China and the United States (8, 72) with almost the same number of children (430 and 408). The studies indicated contradictory associations of BPA concentrations at age 3 years with anthropometric obesity indices at age 7 years. The study from China found positive associations, whereas the US study found null associations, despite the same exposures and outcome ages (8, 72). Many other studies have also reported contradictory findings among the same exposure and outcome age groups (**Tables 1**–**3**). In contrast, some studies conducted in different countries recruiting different populations reported similar associations between the same or different EO and obesity outcomes (7, 34, 62, 82, 83, 89, 92). These conflicting findings across studies might be explained by methodological variations, particularly the characteristics of the study populations. Other potential reasons are exposure levels (low, medium, or high) and the timing and duration of EO exposure. Associations seem to differ between boys and girls, adult males, and females (**Tables 1**–**3**). Some studies reported ethnicity-specific associations between EO exposure and obesity indices (83, 99, 112). The reasons for racial and ethnic differences in overweight and obesity are largely unknown. Possible reasons might be the different patterns of calorie intake or energy consumption, physical activity, metabolic activity, endocrine, and genetic susceptibility among racial and ethnic groups (150, 151).

Among the environmental phenols, BPA has been widely investigated and has been positively associated with anthropometric overweight and obesity indices, mainly in school-aged children, adolescents, and adults. The use of BPA has been decreasing to reduce its negative health impact. This has led to increased use of BPS and BPF. Several studies investigated the association of BPS and BPF with obesity measures and described inconsistent relationships (7, 34, 48, 50, 67). These few studies might be insufficient to conclusively determine the reason for the contradictory associations. Both DEHP and non-DEHP metabolites showed inconsistent associations with overweight and obesity indices at different stages of life. Among the non-DEHP metabolites, MEP, MMP, and MBzP seem to have obesogenic roles in adult and elderly humans. Among the POPs, DDE and PFOA showed almost consistent positive associations with obesity. PFOS also seems to be positively associated with obesity measures, but the associations were sometimes inconsistent. Compared with DDE, DDT showed a weaker association with obesity indices. Although DDT and DDE have already been banned in many countries, the long half-lives of these EOs (7 and 10 years for DDT and DDE, respectively) in both the environment and humans might be responsible for the adverse effects (152–157). Similarly, PFAS metabolites are also very persistent in the environment (half-lives of 3–10 years) and humans are exposed through ingestion of contaminated food, drinking water, and ingestion or inhalation of PFAS from contaminated dust and soil, and even *via* transplacental and breast milk passage from mother to child (158–163).

Usually, a single EO or a group of similar EOs was included in previous studies, making the results straightforward and easily interpretable. The rising concern is that generalized linear regression can provide a simple relationship between a single chemical or a group of similar chemicals and outcomes, but cannot explore the joint effect of mixed exposure (48). In addition, to study causality, researchers need to consider mixed environmental exposures and their complex nonlinear interactions. Eventually, ignoring the joint effects of other chemicals could contribute to false-positive or false-negative results (164). We found only a limited number of studies that investigated the associations between cumulative exposure to EOs and overweight and obesity indices using a multipollutant approach. Findings were inconsistent (48, 103, 116, 117). In one study, the associations of phthalate metabolites and bisphenols with obesity indices varied when considering single and cumulative exposure levels using three different statistical models (48). Thus, the application of a multipollutant statistical model to clarify the joint effects of mixed EOs should be accepted and utilized to explore the effect of a cumulative exposure burden on the outcomes in one direction per occasion, and the exposure-response function of each chemical, while controlling other chemicals at certain levels.

Some EOs (e.g., bisphenols and phthalates) are lipophilic. They can accumulate in adipose tissue of obese women and can influence the development of obesity in their offspring. A recent population-based prospective cohort of 1396 mothers showed that women in highest group of pre-pregnancy BMI (>30kg/m^2^) had significant higher concentrations of BPS [OR=0.15 (0.01, 0.27)] total bisphenols (sum of BPA, BPS, and BPF) [OR= 1.88 (0.13, 4.78)], phthalic acid [OR=13.16 (2.51, 29.86)], high molecular weight phthalate (HMWP) [OR=46.73 (14.56, 93.72)] and DEHP [OR=32.34 (6.90, 70.75)] concentrations in comparison to women in normal pre-pregnancy BMI (20–24.9kg/m^2^) group (165). Another study found that prenatal exposure to PCBs (>1.95 µg/g lipid) was associated with increased BMI in girls from overweight mothers, but not in normal-weight mothers (130). Thus, pre-pregnancy BMI is an important confounder that must be considered when investigating obesity outcomes in a birth cohort. Three studies considered pre-pregnancy BMI as a confounder in birth cohorts (103, 132, 142). Adjustment of pre-pregnancy BMI might shed light on the relationship between EO exposure and obesity.

Daily consumable items (diet or foods and personal care products) are an important route of exposure to several EOs and are intrinsically related to energy balance. BPA and phthalate exposures occur primarily through ingestion and dermal absorption, as these compounds are found in common consumer goods, such as food containers, children’s toys, and personal care products (166–169). Thus, it can be predicted that those who consume or use more of these products are more likely to have higher exposure levels and, perhaps, are more likely to be obese. Several studies reported a direct link between dietary exposure to EOs and obesity (41, 83, 85). Most of the studies in the current review considered diet, calorie intake, energy consumption, and physical activity as potential confounders to address the relationships that strengthen the findings (**Tables 1**–**3**).

Puberty features hormonal transition. Both girls and boys undergo physical changes. Puberty has been associated with the development of obesity (170). Several studies evaluated the relationship between EOs and anthropometric measures of obesity in an age- and sex-specific manner before and after puberty (7, 61, 66, 72, 73, 78, 79, 81, 96). A sex-stratified analyses found that increased exposure to urinary concentrations of BPA was positively associated with the sum of skinfold thickness (ST) in girls, while exposure to MEHP, MEHHP, MECPP, and MEOHP were inversely related to BMI z-score, WC, and the sum of ST in boys (70). However, when the analyze was restricted to children who had not yet begun the pubertal transition, the results shifted and showed positive relationships between BPA in girls and MEHP in boys with the sum of ST. In the prenatal exposure period, the authors observed an inverse relationship between MBzP and a child’s BMI z-score, but this finding did not persist when the analyses were restricted to children prior to puberty. In a case-control study, prepubertal girls showed positive associations between %MEHHP and BMI, WC, and %BF, and showed significantly increased odds in the 3^rd^ and 4^th^ quartiles compare to the 1^st^ quartile, whereas the relationship was null in pubertal girls (90). How the associations differ before and after puberty is not yet clearly understood. Knowledge of hormone levels related to pubertal growth, including thyroid hormones, leptin, adiponectin, and others, might provide more insight into the potential mechanisms of EO-mediated s adiposity (171).

### Research Gap

One of the limitations of the birth cohort studies outlined here is the use of single spot urine during the 1^st^, 2^nd,^ or 3^rd^ trimester to estimate EO exposure. The biological half-lives of some of these chemicals are short and they are quickly excreted in urine (e.g., phthalates and bisphenols). Epidemiologists ideally prefer to use 24 h urine and repeated urine sampling when assessing these chemicals in relation to obesity, which occurs incrementally over time and has a multifactorial etiology (172, 173). The time of the day or season could account for some intrapersonal or interpersonal variations in urinary concentrations of analytes in single spot urine samples (174–177). However, single spot is the conventional test, despite these methodological limitations. In most of the included studies, biomonitoring EOs were done using methods lacking validated external quality assurance. Maintaining internal and external quality control and quality assurance might make study findings comparable and could strengthen the findings. Some studies had very limited information on pre-pregnancy BMI due to the availability of self-reported weight and the timing of recruitment in their original birth cohorts. These studies relied on maternal BMI. However, most of the studies collected data using self-reported questionnaires or home visits. Therefore, under- or over-estimated data could not be avoided. There was little or no data of phthalate metabolite (both DEHP and non-DEHP) levels and subsequent obesity assessment in infants and toddlers (**Matrix Tables 2**, **3**). In addition, the assessment of obesity in adolescents related to OC (DDT, DDE, β-HCH, HCB, and PCBs) exposure was insufficient (**Matrix Table 6**). Very few studies investigated the relationship between cumulative EO exposure levels and overweight or obesity indices. Therefore, the possibility that prenatal and/or postnatal exposure to other unmeasured chemicals correlated with measured chemicals may have confounded the associations under study cannot be excluded. Finally, there are scant data concerning the trajectories of exposure and outcome assessments.

### Future Contemplations and Research Design

Environmental epidemiologists should clearly infer whether exposure to ECs might influence weight gain or obesity, or whether obese study participants might have greater exposure to, or excretion of, ECs by conducting long-term follow-up studies in child and adult populations. Further prospective studies should aim to collect data with repeated measures over extended periods to improve exposure classification, increase general understanding of the timing of exposure, and address the temporal relationship between ECs and obesity. Given the gradual decrease of some ECs and increase exposure to some alternate ECs in human populations, continued biomonitoring of these alternate ECs and further investigations on their obesogenic effects in humans could be undertaken. Researchers should target study participants at all stages of life to assess exposure and obesity outcomes at each age. Many other chemicals, including pesticides, heavy metals, and particulate matter, have been reportedly associated with obesity outcomes *in vitro* and *in vivo* in animal studies. However, their obesogenic effects have not yet been completely evaluated in humans (178–186). Thus, an exposome-based approach needs to be developed to investigate the possible obesogenic effects of chemicals, xenobiotics, and pollutants in humans to explore the overall scenario of cumulative exposure. Studies of the obesogenic effects of ECs in the context of diet, stress, ethnicity, gender, and other factors, using sophisticated statistical models to assess complex exposures should be done.

## Conclusions

The collective data indicate that BPA, DDE, and PFOA have consistent obesogenic effects in humans. Other bisphenols, phthalates, and POP metabolites or congeners have contradictory relationships with obesity at different outcome assessment times. Further prospective cohort studies with cumulative exposure assessments are required. The findings of this review will increase the awareness of the obesogenic effects of ECs among the general population.

## Author Contributions

NCM: investigation, data curation, analysis, and writing-original draft. SK: data curation and writing–review and editing. YI and MK: conceptualization, project administration, funding acquisition, supervision, and writing–review and editing. All authors contributed to the article and approved the submitted version.

## Funding

This research was conducted with the support of the Grants-in-Aid for Scientific Research 19H01078 and 19H03888 provided by the Japan Society for the Promotion of Science (JSPS).

## Conflict of Interest

The authors declare that the research was conducted in the absence of any commercial or financial relationships that could be construed as a potential conflict of interest.

## Publisher’s Note

All claims expressed in this article are solely those of the authors and do not necessarily represent those of their affiliated organizations, or those of the publisher, the editors and the reviewers. Any product that may be evaluated in this article, or claim that may be made by its manufacturer, is not guaranteed or endorsed by the publisher.

## Abbreviations

BF, body fat; BMI, body mass index; BPA, bisphenol A; BPF, bisphenol F; BPS, bisphenol S; β-HCH, beta-hexachlorocyclohexane; DBP, dibutyl phthalate; DDT, dichlorodiphenyltrichloroethane; DDE, dichlorodiphenyldichloroethylene; DEHP; di(2-ethylhexyl) phthalate; EC, environmental chemical; EO, environmental obesogen; FM, fat mass; HC, hip circumference; HCB, hexachlorobenzene; HMWP, high molecular weight phthalate; LMWP, low molecular weight phthalate; MBP, mono-butyl phthalate/mono-n-butyl phthalate; MBzP, mono-benzyl phthalate; MCMHP, mono-2-carboxymethyl-hexyl phthalate; MCPP, mono-(3-carboxypropyl) phthalate; MEP, mono-ethyl phthalate; MEHP, mono-2-ethylhexyl phthalate; MEOHP, mono (2-ethyl-5-exohexyl) phthalate; MEHHP, mono (2-ethyl-5hydroxyhexyl) phthalate; MEHP, mono-ethylhexyl-phthalate; MECPP, mono-(2-ethyl-5-carboxypentyl) phthalate; MiBP, mono-isobutyl phthalate; MMP, mono-methyl phthalate; OC, organochlorine compound; PBDEs, polybrominated diphenyl ethers; PCB, polychlorinated biphenyl; PFAS, per- and polyfluoroalkyl substances; POPs, persistent organic pollutants; ST, skinfold thickness; WC, waist circumference; WHtR, waist-to-height ratio; WtHR, weight-to-height ratio.
